# Assessment of Local and Systemic Changes in Plant Gene Expression and Aphid Responses during Potato Interactions with Arbuscular Mycorrhizal Fungi and Potato Aphids

**DOI:** 10.3390/plants9010082

**Published:** 2020-01-09

**Authors:** Eric Rizzo, Tyler Sherman, Patricia Manosalva, S. Karen Gomez

**Affiliations:** 1 School of Biological Sciences, University of Northern Colorado, 501 20th St., Greeley, CO 80639, USA; ericrizzo411@gmail.com (E.R.); tsherman11@gmail.com (T.S.); 2 Department of Microbiology and Plant Pathology, University of California, Riverside, 900 University Avenue, Riverside, CA 92521, USA; pmanosal@ucr.edu

**Keywords:** arbuscular mycorrhizal symbiosis, local responses, potato aphids, plant gene expression, potato, systemic responses

## Abstract

This research examined aphid and plant responses to distinct levels (none, low, and high) of arbuscular mycorrhizal (AM) fungal root colonization by studying the association between potato aphids (*Macrosiphum euphorbiae*), potatoes (*Solanum tuberosum*), and AM fungi (*Rhizophagus intraradices*). It extends knowledge on gene expression changes, assessed by RT–qPCR, of ten defense-related genes at two time-points post-herbivory (24 h and 10 days), focusing on aphid-infested local leaves, non-infested systemic leaves, and roots. The results showed that aphid fitness was not altered by AM symbiosis. At 24 h, *ETHYLENE RECEPTOR* *1* gene expression was repressed in roots of aphid-infested non-mycorrhizal plants and aphid-infested plants with a high level of AM fungal root colonization, but not on aphid-infested plants with a low level of AM fungal root colonization. At 10 days, *ALLENE OXIDE CYCLASE* and *POTATO TYPE I PROTEASE INHIBITOR* were upregulated exclusively in local leaves of aphid-infested plants with a low level of AM fungal root colonization. In addition, local and systemic changes in plant gene expression appeared to be regulated exclusively by AM status and aphid herbivory. In summary, the gene expression data provide insights on mycorrhizal potato responses to aphid herbivory and serve as a starting point for future studies using this system.

## 1. Introduction

Arbuscular mycorrhizal (AM) fungi are mostly known for aiding plants with the uptake of soil nutrients, such as phosphorus [1,2], and for helping plants better cope with abiotic [2,3,4,5] and biotic [2,6,7,8,9,10,11] stress. Another aspect of this ubiquitous symbiosis that is intriguing involves the impact of AM fungi on plant immunity, especially against aboveground and belowground insect herbivores [12,13,14]. Beneficial soil microbes such as AM fungi are able to alter plant–insect herbivore interactions through several mechanisms, eliciting changes in the availability and quality of plant nutrients, defensive strategies, and stress tolerance [15]. In addition, AM fungi can affect insect herbivores aboveground, both by changing host-plant quality from the bottom-up [13,16,17] and the resistance of insect herbivores to their natural enemies from the top-down [12,18,19]. During the early developmental stages of the symbiosis, plant defense responses are modulated to facilitate AM fungal colonization of roots, which then leads to activation of plant immune responses both at the local level and throughout the plant [17]. Thus, this activation contributes to “priming”, wherein subsequent attacks by pathogens or insect herbivores are met with a more efficient activation of plant defenses [17,20]. The plant hormone jasmonic acid (JA) has been proposed as a major contributor in boosting the plant’s immune system, resulting in mycorrhiza-induced resistance (MIR) [17,21,22]. However, research has established that the general extent of MIR against arthropod herbivores is highly dependent upon the level of specialization and the modes of feeding of the attacking insect [14,16,23,24,25]. A meta-analysis showed that AM fungi negatively impact the performance of generalist chewing herbivores, yet yield positive effects on specialist chewing insects [16]. The present study provides insights into the interaction among AM fungi, plants, and phloem feeders such as aphids. 

The response of aphids to AM fungal colonization of their host plants varies widely, from positive [24,26,27,28,29,30,31] to neutral [13,14,32,33,34,35] or negative [27,29,30,36,37,38,39]. In general, specialist and generalist aphids benefit from feeding on mycorrhizal plants [16,24,26,27,28,29,30,39], while specialist chewing herbivores, such as caterpillars, are negatively affected by AM fungal colonization of their host plants [13,16]. It is possible that phloem feeders may benefit from feeding on AM fungus-colonized host plants because of the mycorrhiza-induced enhancement of plant vascular bundle size [28,40]. Moreover, improved plant quality may benefit grain aphids (*Sitobion avenae* Fabricius) feeding on mycorrhizal barley (*Hordeum vulgare* L.) plants by acquiring nitrogen [41]. In summary, previous studies found that aphids are more attracted to mycorrhizal plants, gain body mass, and show increased relative growth rates and fecundity on host plants colonized by AM fungi compared to non-mycorrhizal plants [24,26,27,28,29,34,42]. In previous studies, the extent of AM fungal root colonization ranged between <10% (arbuscules only) and 74% root length colonized (RLC), which may in part explain some of the variation in aphid responses to root colonization by AM fungi.

Currently, a limited number of studies involving aphid–plant–AM fungi interactions used more than one level of AM fungal root colonization [27,29,30,43]. In most studies, non-mycorrhizal plants were compared to plants whose roots were either highly colonized or low-colonized by AM fungi. A study that included more than one level of AM fungal root colonization found that pea aphids (*Acyrthosiphon pisum* Harris) grew faster if broad bean (*Vicia faba* L.) plants were already colonized by AM fungi (40%–60% RLC) at the beginning of aphid feeding, compared with insects on non-mycorrhizal plants [27]. However, pea aphids grew slower after feeding on plants that were colonized with AM fungi (20%–40% RLC) after aphid infestation [27]. The effects of AM symbiosis on aphid performance depended on a combination of the AM developmental stage and plant age [43]. The reduction in the relative growth rate of the green peach aphid (*Myzus persicae* Sulzer) was observed when insects fed on young *Plantago lanceolata* L. plants with low levels of AM fungal root colonization (10% RLC), whereas aphids benefited when feeding on older plants with high levels of AM fungal root colonization (80% RLC) [43]. It was shown recently that pea aphid colony weight increased after feeding for seven days on *Medicago truncatula* (Gärtner) plants that have high levels of AM fungal colonization of roots (58%–74% RLC) when compared to non-mycorrhizal controls [29]. Additionally, when given a choice, pea-aphid adults showed a clear preference for plants having high levels of AM fungal root colonization compared to non-mycorrhizal controls [29]. Finally, the mass and growth rate of the specialist Oleander aphid (*Aphis nerii* Boyer de Fonscolombe) increased after feeding on milkweed (*Asclepias*) plants that have high levels of AM fungi inoculum when compared to medium levels of AM fungi inoculum [30]. These trends seem to indicate that aphids mostly benefit from feeding on plants whose roots are highly colonized by AM fungi, while the opposite is observed on plants whose roots are at low levels of colonization. Nevertheless, the molecular mechanisms that drive this variation in aphid responses to AM fungi remains elusive.

The present study was designed to examine aphid and plant responses to distinct levels of AM fungal colonization of roots by studying the interaction between the potato aphid *Macrosiphum euphorbiae* (Thomas), potato *Solanum tuberosum* L. cv. Désirée, and the generalist AM fungus *Rhizophagus intraradices* (N.C. Schenck and G.S. Sm.) C. Walker and A. Schüßler. It investigates whether low and high levels of AM fungal root colonization impact aphid herbivory and measures changes in the expression of several plant genes. In this study, plant age was the same, but the level of AM fungus inoculum was varied. To date, assessment of plant gene expression focusing on leaves damaged by aphids (local leaf), undamaged leaves (systemic leaf), and roots during aphid–plant–AM fungi interactions is lacking. Localized and systemic induced resistance conferred by AM fungi has been reported against root pathogens [10] and root nematodes [44], while local and systemic susceptibility triggered by AM fungi to different pests has been reported in rice [45]. The research objectives of this study were to (a) examine the indirect effect of AM fungal root colonization on potato aphid–potato interactions, (b) measure plant gene expression and determine whether mycorrhizal potato plants having low and high levels of AM fungal root colonization show altered transcript levels of defense-related genes, and (c) investigate whether aphid-damaged leaves and/or systemic undamaged leaves exhibit priming following AM fungal root colonization. The following hypotheses were tested in this study: (1) aphids would show decreased aphid fitness after feeding for 10 days on potato plants with a low level of AM fungal root colonization; (2) aphids would show improved aphid fitness after feeding for 10 days on potato plants with a high level of AM fungal root colonization; and (3) potato-aphid-infested plants with a low level of AM fungal root colonization would show an increase in levels of defense-related transcripts in local and/or systemic leaves when compared with the levels in potato-aphid-infested non-mycorrhizal plants and plants with high levels of AM fungal root colonization.

## 2. Results

### 2.1. Impact of Two Levels of AM Fungal Root Colonization on Potato Aphid Fitness

**Figure 1:**
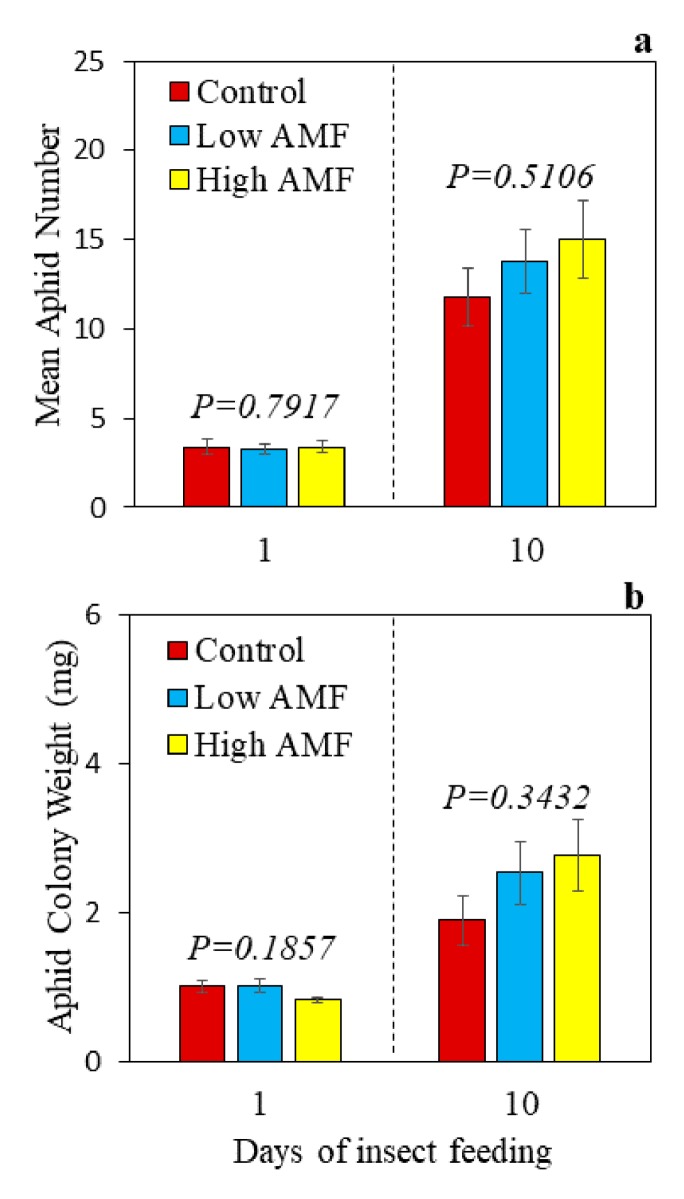
Potato aphid (*Macrosiphum euphorbiae*) (**a**) number per colony (per plant) and (**b**) weight per colony after one and ten days of herbivory on non-mycorrhizal potato plants (*Solanum tuberosum*) (Control), plants with a low level of AM fungal root colonization (Low AMF), and plants with a high level of AM fungal root colonization (High AMF). Values represent the mean of seven biological replicates ± SEM. A *p* ≥ 0.05 indicates no statistical significance based on ANOVA.

This research tested whether aphid fitness, measured as insect weight and abundance, changes when aphids feed on plants that have different levels of AM fungal root colonization. In this study, the aphid colony weight and the mean aphid number per colony did not change in any of the treatments, indicating that potato aphid fitness was not altered by root colonization with AM fungi (Figure 1). The data showed that AM fungal root colonization was significantly higher in plants that received a more concentrated inoculum (High AMF) compared to plants that received a more diluted inoculum (Low AMF), both at 24 h and 10 days post-aphid-feeding, independent of aphid infestation (Figure S1), indicating that the two distinct levels were successfully achieved. Moreover, colonization levels were higher at 10 days than 24 h for all the treatments, as expected (Figure S1). Furthermore, potato shoot and root fresh weights were not altered by AM fungal root colonization at the time-points tested (Figures S2 and S3).

### 2.2. Plant Gene Expression after 24 h of Potato Aphid Herbivory at Two Levels of AM Fungal Root Colonization

**Figure 2:**
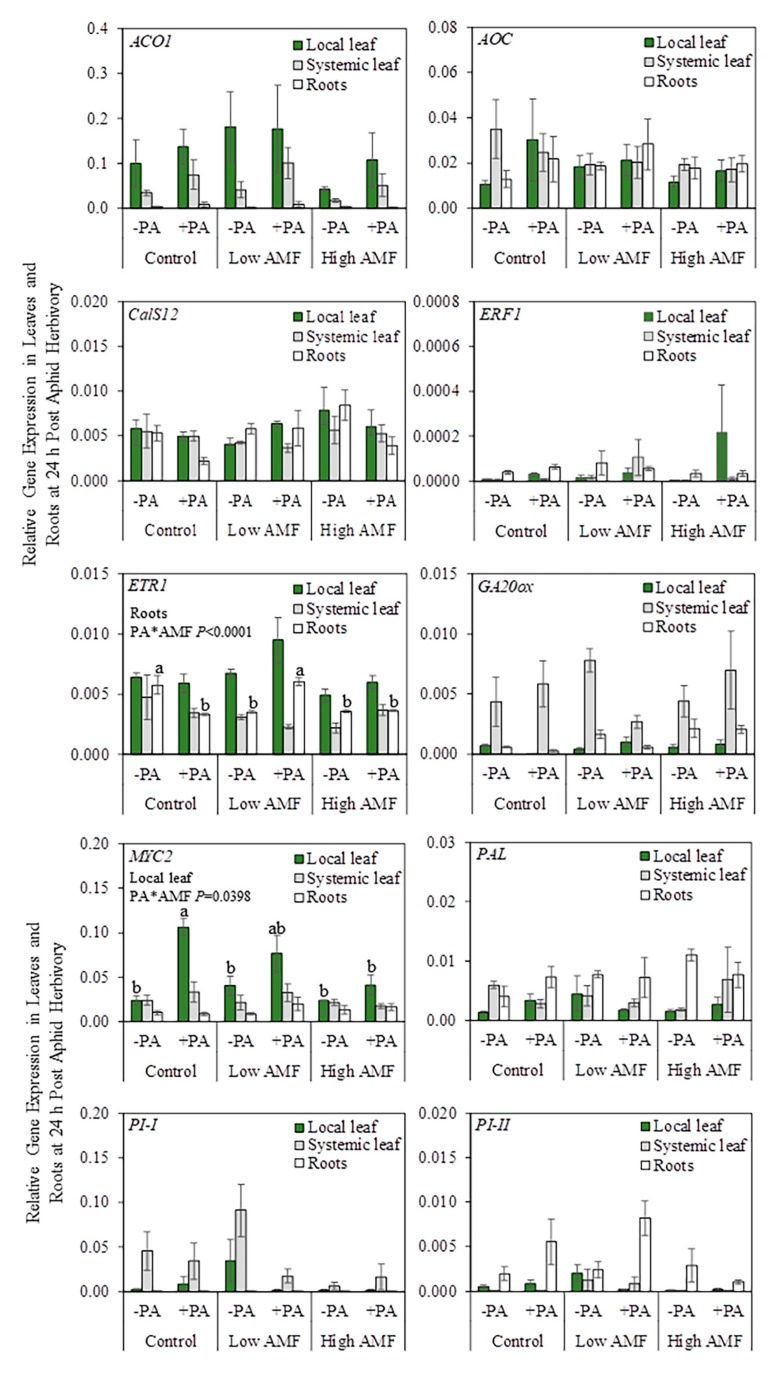
Relative gene expression in local leaf, systemic leaf, and roots after 24 h of herbivory by potato aphids (*Macrosiphum euphorbiae*) on potato plants (*Solanum tuberosum*). Non-infested non-mycorrhizal plants (−PA Control); potato-aphid-infested non-mycorrhizal plants (+PA Control); non-infested plants with a low level of AM fungal root colonization (−PA Low AMF); potato-aphid-infested plants with a low level of AM fungal root colonization (+PA Low AMF); non-infested plants with a high level of AM fungal root colonization (−PA High AMF); and potato-aphid-infested plants with a high level of AM fungal root colonization (+PA High AMF). The only PA*AMF interactions that were significant in the two-factor ANOVA were for *ERT1* (roots) and *MYC2* (local leaf). One-factor ANOVA followed by the Tukey–Kramer test was used when the interaction term was significant (PA*AMF *p* ≤ 0.05). Different letters indicate significant difference among treatments based on the Tukey–Kramer test (*p* ≤ 0.05). Values represent the mean ± SEM of three biological replicates and two technical replicates per treatment. The *p*-values from Table S1 were used to generate this figure. *ACO1 = AMINOCYCLOPROPANE-1-CARBOXYLATE OXIDASE 1; AOC = ALLENE OXIDE CYCLASE; CalS12 = CALLOSE SYNTHASE 12; ERF1 = ETHYLENE RESPONSE FACTOR 1; ETR1 = ETHYLENE RECEPTOR 1; GA20ox = GIBBERELIC ACID 20-OXIDASE; MYC2 = BASIC-HELIX-LOOP-HELIX TRANSCRIPTION FACTOR; PAL = PHENYLALANINE AMMONIA LYASE; PI-I = POTATO TYPE I PROTEASE INHIBITOR; PI-II = POTATO TYPE II PROTEASE INHIBITOR*.

This experiment aimed to do the following: (a) measure plant gene expression to determine whether mycorrhizal potato plants that have low and high levels of AM fungal root colonization show altered levels of defense-related transcripts, and (b) investigate whether aphid-damaged leaves and/or systemic undamaged leaves exhibit priming following colonization of roots by AM fungi. From the ten defense-response genes tested that are associated with SA, JA, and ET pathways, *ETR1* and *MYC2* gene expression changed in roots and local leaves, respectively, during the interaction between potato aphid herbivory and AM fungal root colonization (Figure 2 and Table S1. In roots, *ETR1* gene expression was downregulated in potato-aphid-infested non-mycorrhizal plants (+PA Control), non-infested plants with a low level of AM fungal root colonization (−PA Low AMF), and both non-infested and potato-aphid-infested plants with a high level of AM fungal root colonization (−PA High AMF and +PA High AMF) when compared with the levels in non-infested non-mycorrhizal plants (−PA Control) and potato-aphid-infested plants with a low level of AM fungal root colonization (+PA Low AMF). In local leaves, *MYC2* gene expression was upregulated in potato-aphid-infested non-mycorrhizal plants (+PA Control) compared to the levels in non-infested non-mycorrhizal plants (−PA Control), non-infested plants with a low level of AM fungal root colonization (−PA Low AMF), and both non-infested and potato-aphid-infested plants with a high level of AM fungal root colonization (−PA High AMF and +PA High AMF). *MYC2* gene expression in potato-aphid-infested plants with a low level of AM fungal root colonization (+PA Low AMF) was not different from any other treatment (Figure 2).

Overall, the interaction between potato aphid herbivory and AM fungal root colonization did not have a significant effect (PA*AMF *p* ≥ 0.05) on the genes tested in local leaves, systemic leaves, and in roots, with the exception of *ETR1* and *MYC2*, under the conditions tested in this study (Figure 2 and Table S1).

**Figure 3:**
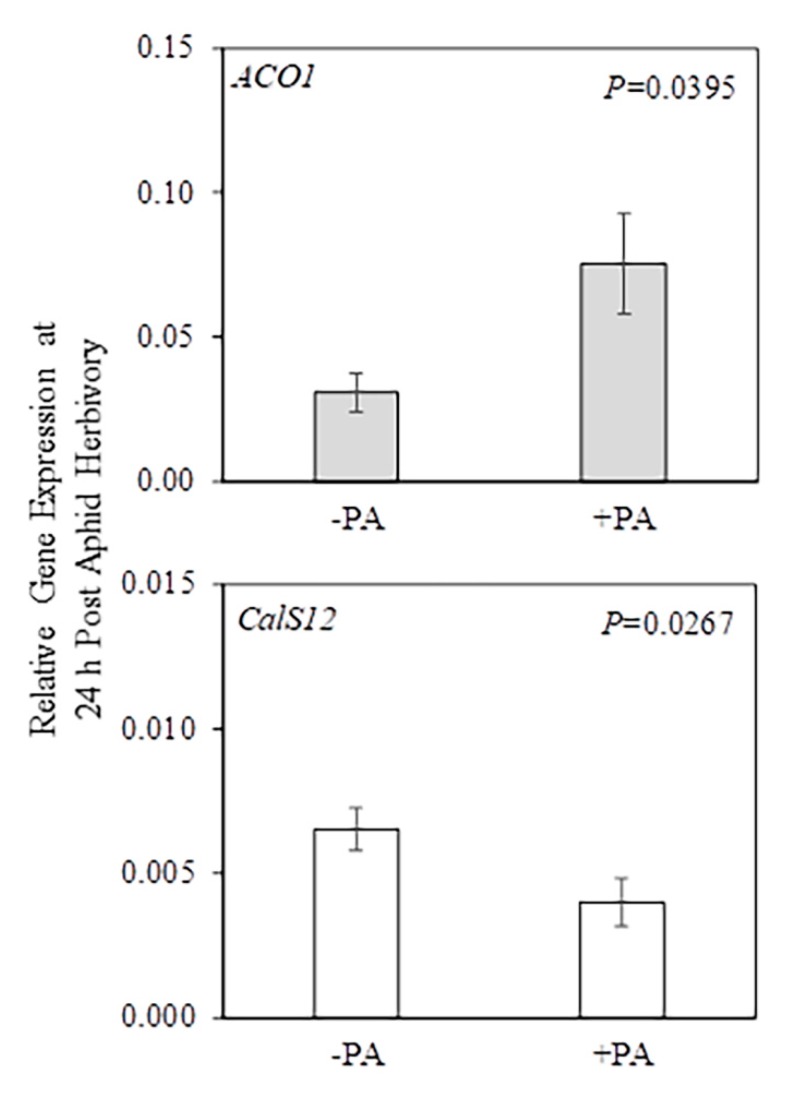
Relative gene expression in systemic leaf (gray bar) and roots (white bar) after 24 h herbivory by potato aphids (*Macrosiphum euphorbiae*) on potato plants (*Solanum tuberosum*). The PA*AMF interaction was not significant in the two-factor ANOVA, but potato aphid herbivory as a main factor had a significant effect (PA *p* ≤ 0.05) on *ACO1* and *CalS12* gene expression. Values represent the mean ± SEM (n = 9 for −PA/+PA). The *p*-values from Table S1 were used to generate this figure. PA = potato aphid. *ACO1 = AMINOCYCLOPROPANE-1-CARBOXYLATE OXIDASE 1; CalS12 = CALLOSE SYNTHASE 12*.

The data also showed that potato aphid herbivory as a main factor had a significant (PA *p* ≤ 0.05) effect on *ACO1* gene expression in systemic leaves and the *CalS12* gene expression in roots (Figure 3 and Table S1), indicating that these genes are regulated by potato aphid herbivory whether plants were colonized or not by AM fungi. In systemic leaves, the mean gene expression of *ACO1* in potato-aphid-infested plants (+PA Control, +PA Low AMF, and +PA High AMF) was higher when compared with the mean of non-infested plants (−PA Control, −PA Low AMF, and −PA High AMF). Meanwhile, the mean gene expression of *CalS12* in roots was significantly higher in non-infested plants (−PA Control, −PA Low AMF, and −PA High AMF) when compared with the mean of potato-aphid-infested plants (+PA Control, +PA Low AMF, and +PA High AMF) (Figure 3).

**Figure 4:**
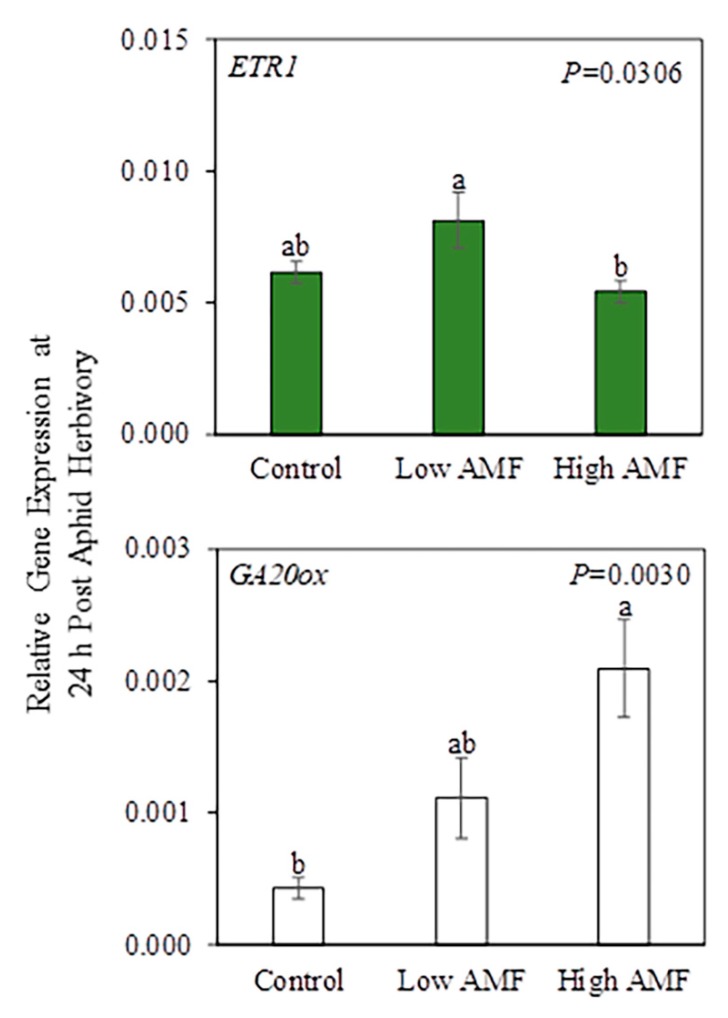
Relative gene expression in local leaf (green bar) and roots (white bar) after 24 h herbivory by potato aphids (*Macrosiphum euphorbiae*) on potato plants (*Solanum tuberosum*). The PA*AMF interaction was not significant in the two-factor ANOVA, but AM fungal root colonization as a main factor had a significant effect (AMF *p* ≤ 0.05) on *ETR1* and *GA20ox* gene expression. Values represent the mean ± SEM (n = 6 for Control/Low AMF/High AMF). The *p*-values from Table S1 were used to generate this figure. Different letters indicate significant difference among groups based on the Tukey–Kramer test (*p ≤* 0.05). Control = non-mycorrhizal plants; Low AMF = plants with a low level of AM fungal root colonization; High AMF = plants with a high level of AM fungal root colonization. *ETR1 = ETHYLENE RECEPTOR 1; GA20ox = GIBBERELIC ACID 20-OXIDASE*.

AM fungal root colonization as a main factor significantly (AMF *p* ≤ 0.05) impacted the level of *ETR1* transcripts in local leaves and *GA20ox* in roots (Figure 4 and Table S1), indicating that these genes are differentially regulated by AM fungal colonization of roots whether plants were damaged or not by potato aphids.

In local leaves, the mean gene expression of *ETR1* in non-mycorrhizal plants (−PA Control and +PA Control) did not differ from the level in mycorrhizal plants (−PA Low AMF, +PA Low AMF, −PA High AMF, and +PA High AMF) (Figure 4); however, the mean gene expression of *ETR1* was higher in plants with a low level of AM fungal root colonization (−PA Low AMF and +PA Low AMF) compared to the level in plants with a high level of AM fungal root colonization (−PA High AMF and +PA High AMF), suggesting that a high level of root colonization by AM fungi suppresses *ETR1* gene expression. In roots, plants with a high level of AM fungal root colonization (−PA High AMF and +PA High AMF) exhibited upregulation of *GA20ox* gene expression when compared with non-mycorrhizal plants (−PA Control and +PA Control). The mean gene expression of *GA20ox* in plants with a low level of AM fungal root colonization (−PA Low AMF and +PA Low AMF) was not different from those in the other treatments (Figure 4).

### 2.3. Plant Gene Expression after 10 Days of Potato Aphid Herbivory at Two Levels of AM Fungal Root Colonization

**Figure 5:**
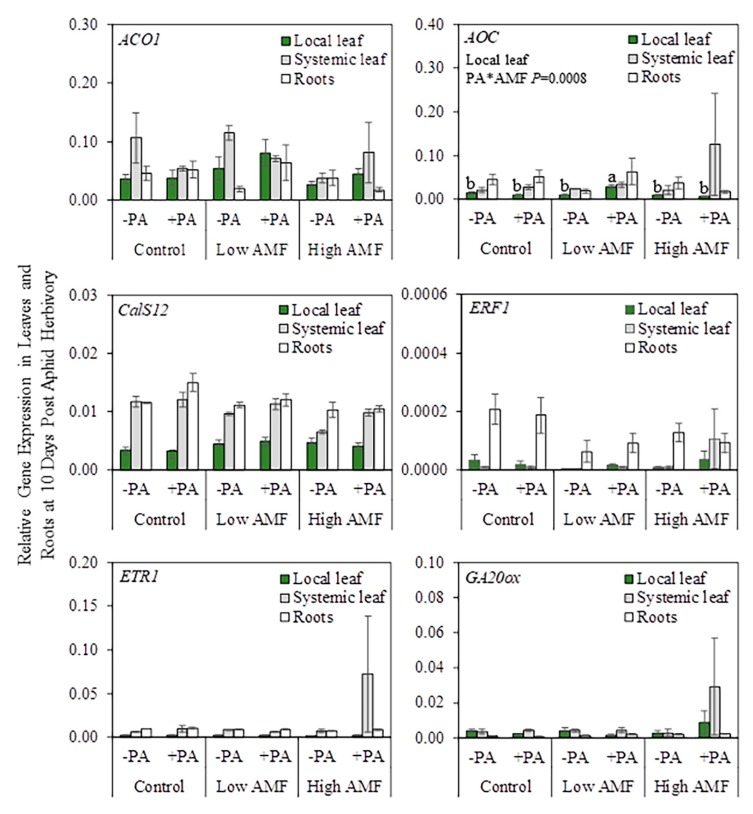

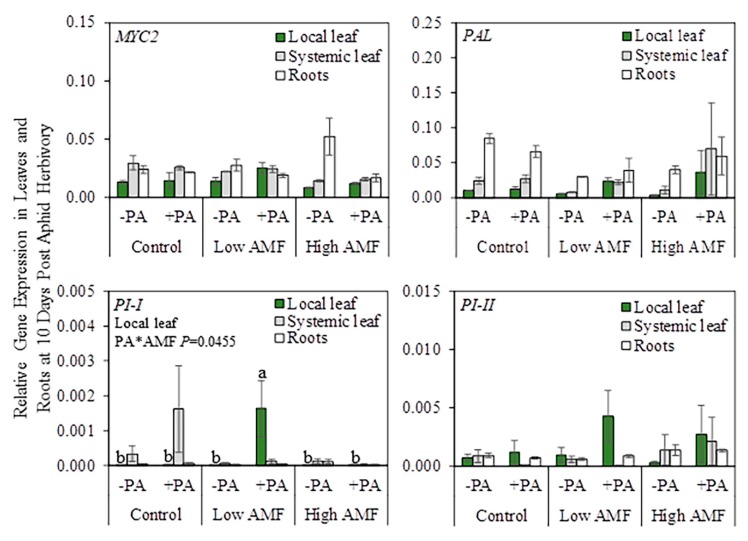
Relative gene expression in local leaf, systemic leaf, and roots after 10 days of herbivory by potato aphids (*Macrosiphum euphorbiae*) on potato plants (*Solanum tuberosum*). Non-infested non-mycorrhizal plants (−PA Control); potato-aphid-infested non-mycorrhizal plants (+PA Control); non-infested plants with a low level of AM fungal root colonization (−PA Low AMF); potato-aphid-infested plants with a low level of AM fungal root colonization (+PA Low AMF); non-infested plants with a high level of AM fungal root colonization (−PA High AMF); and potato-aphid-infested plants with a high level of AM fungal root colonization (+PA High AMF). The only PA*AMF interactions that were significant (PA*AMF *p* ≤ 0.05) in the two-factor ANOVA were for *AOC* and *PI-I* in local leaves. One-factor ANOVA followed by the Tukey-Kramer test was used when the interaction term was significant. Different letters indicate significant difference among treatments based on the Tukey–Kramer test (*p* ≤ 0.05). Values represent the mean ± SEM of three biological replicates and two technical replicates per treatment. The *p*-values from Table S2 were used to generate this figure. *ACO1 = AMINOCYCLOPROPANE-1-CARBOXYLATE OXIDASE 1; AOC = ALLENE OXIDE CYCLASE; CalS12 = CALLOSE SYNTHASE 12; ERF1 = ETHYLENE RESPONSE FACTOR 1; ETR1 = ETHYLENE RECEPTOR 1; GA20ox = GIBBERELIC ACID 20-OXIDASE; MYC2 = BASIC-HELIX-LOOP-HELIX TRANSCRIPTION FACTOR; PAL = PHENYLALANINE AMMONIA LYASE; PI-I = POTATO TYPE I PROTEASE INHIBITOR; PI-II = POTATO TYPE II PROTEASE INHIBITOR*.

This experiment aimed to (a) measure plant gene expression to determine whether mycorrhizal potato plants with low and high levels of AM fungal root colonization showed altered levels of defense-related transcripts and (b) investigate whether aphid-infested leaves and/or systemic undamaged leaves exhibit priming following the colonization of roots by AM fungi. The interaction between potato aphid herbivory and AM fungal root colonization had a significant effect (PA*AMF *p* ≤ 0.05) on the expression of the genes *AOC* and *PI-I*, primarily in local leaves at 10 days post-aphid-feeding (Figure 5 and Table S2).

*AOC* and *PI-I* gene expression in local leaves was higher in potato-aphid-infested plants with a low level of AM fungal root colonization (+PA Low AMF) compared to the levels in all other treatments (−PA Control, +PA Control, −PA Low AMF, −PA High AMF, and +PA High AMF). 

**Figure 6:**
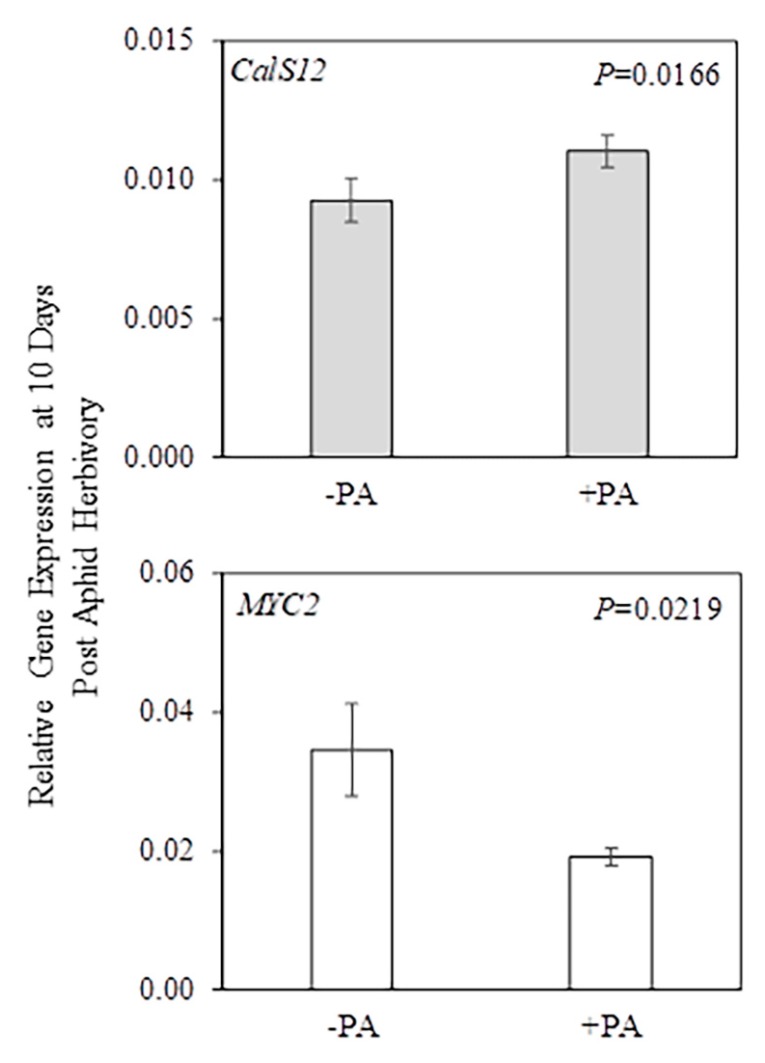
Relative gene expression in systemic leaf (gray bar) and roots (white bar) after 10 days of herbivory by potato aphids (*Macrosiphum euphorbiae*) on potato plants (*Solanum tuberosum*). The PA*AMF interaction was not significant in the two-factor ANOVA, but potato aphid herbivory as a main factor had a significant effect (PA *p* ≤ 0.05) on *CalS12* and *MYC2* gene expression. Values represent the mean ± SEM (n = 9 for −PA/+PA). The *p*-values from Table S2 were used to generate this figure. PA = potato aphid. *CalS12 = CALLOSE SYNTHASE 12; MYC2 = BASIC-HELIX-LOOP-HELIX TRANSCRIPTION FACTOR*.

Potato aphid herbivory as a main factor significantly (PA *p* ≤ 0.05) effected *CalS12* gene expression in systemic leaves and *MYC2* gene expression in roots (Figure 6 and Table S3), indicating that these genes are regulated by potato aphid herbivory whether plants were colonized or not by AM fungi. The *CalS12* gene was differentially expressed at 24 h (*CalS12* in roots; Figure 3) and 10 days (*CalS12* in systemic leaves; Figure 6). At 10 days post-aphid-herbivory, *CalS12* transcript levels in systemic leaves were higher in potato-aphid-infested plants (+PA Control, +PA Low AMF, and +PA High AMF) compared to the levels in non-infested plants (−PA Control, −PA Low AMF, and −PA High AMF). In contrast, *MYC2* transcript levels decreased in roots of aphid-infested plants (+PA Control, +PA Low AMF, and +PA High AMF) compared to non-infested plants (−PA Control, −PA Low AMF, and −PA High AMF).

**Figure 7:**
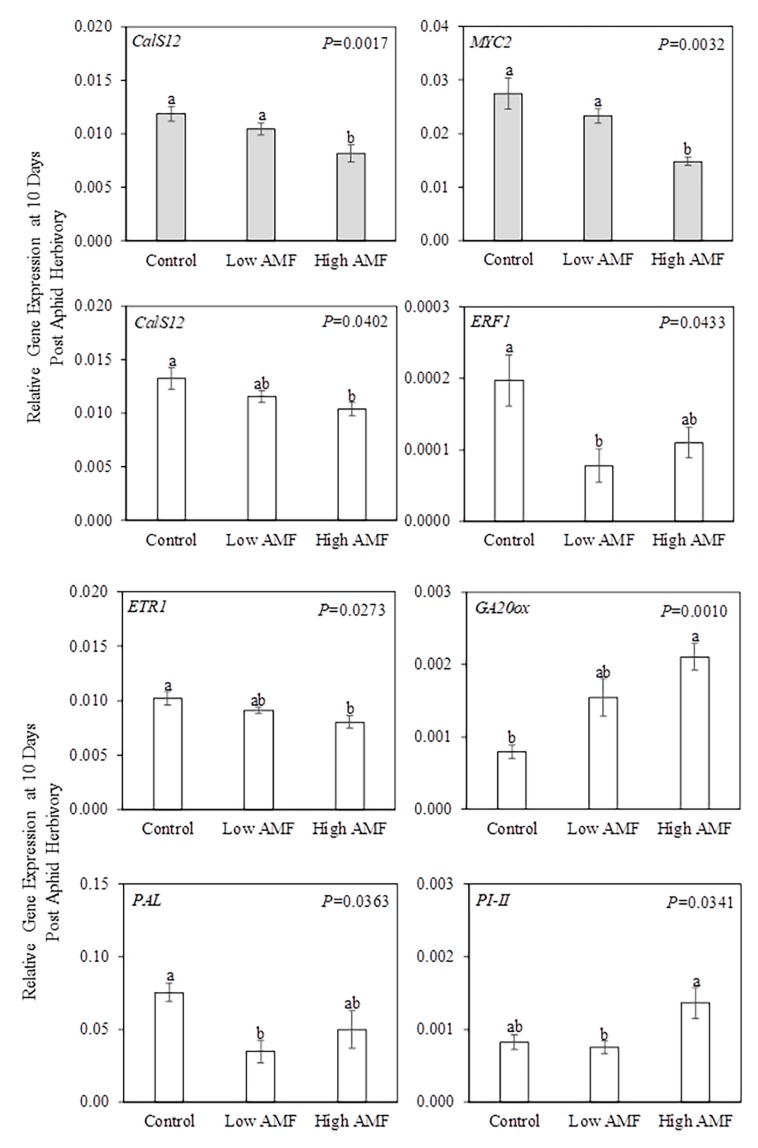
Relative gene expression in systemic leaf (gray bar) and roots (white bar) after 10 days of herbivory by potato aphids (*Macrosiphum euphorbiae*) on potato plants (*Solanum tuberosum*). The PA*AMF interaction was not significant in the two-factor ANOVA, but AM fungal root colonization as a main factor had a significant effect (AMF *p* ≤ 0.05) on *CalS12*, *MYC2*, *ERF1*, *ETR1*, *GA20ox*, *PAL,* and *PI-II* gene expression. Values represent the mean ± SEM (n = 6 for Control/Low AMF/High AMF). The *p*-values from Table S2 were used to generate this figure. Different letters indicate significant difference among groups based on the Tukey–Kramer test (*p ≤* 0.05). Control = non-mycorrhizal plants; Low AMF = plants with a low level of AM fungal root colonization; High AMF = plants with a high level of AM fungal root colonization. *CalS12 = CALLOSE SYNTHASE 12; ERF1 = ETHYLENE RESPONSE FACTOR 1; ETR1 = ETHYLENE RECEPTOR 1; GA20ox = GIBBERELIC ACID 20-OXIDASE; MYC2 = BASIC-HELIX-LOOP-HELIX TRANSCRIPTION FACTOR; PAL = PHENYLALANINE AMMONIA LYASE; PI-II = POTATO TYPE II PROTEASE INHIBITOR*.

The AM fungal root colonization as a main factor significantly (AMF *p* ≤ 0.05) effected gene expression of the genes *CalS12* and *MYC2* in systemic leaves, and six genes in roots including *CalS12*, *ERF1*, *ETR1*, *GA20ox*, *PAL*, and *PI-II* (Figure 7, Table S2), indicating that these genes are regulated by AM status whether plants were damaged or not by potato aphids.

In systemic leaves, *CalS12* and *MYC2* shared the same pattern of gene expression, showing that plants with a high level of AM fungal root colonization (−PA High AMF and +PA High AMF) had reduced levels of transcripts when compared with the levels in non-mycorrhizal plants (−PA Control and +PA Control) and plants with a low level of AM fungal root colonization (−PA Low AMF and +PA Low AMF) (Figure 7). In roots, *CalS12* and *ETR1* shared the same pattern of gene expression, showing that plants with a high level of AM fungal root colonization (−PA High AMF and +PA High AMF) had reduced levels of transcripts of both genes compared to the mean gene expression level in non-mycorrhizal plants (−PA Control and +PA Control); in contrast, the mean gene expression in plants with a low level of AM fungal root colonization (−PA Low AMF and +PA Low AMF) did not differ from the other treatments. Moreover, *ERF1* and *PAL* shared the same pattern of gene expression in roots, showing that both genes had reduced levels of transcripts in plants with a low level of AM fungal root colonization (−PA Low AMF and +PA Low AMF) compared to the gene expression levels in non-mycorrhizal plants (−PA Control and +PA Control); meanwhile, gene expression levels in plants with a high level of AM fungal root colonization (−PA High AMF and +PA High AMF) did not differ from the other treatments. Plants with a high level of AM fungal root colonization (−PA High AMF and +PA High AMF) showed upregulation of *GA20ox* gene expression when compared to non-mycorrhizal plants (−PA Control and −PA Control); however, gene expression in plants with a low level of AM fungal root colonization (−PA Low AMF and +PA Low AMF) did not differ from the other treatments. Finally, plants with a low level of AM fungal root colonization (−PA Low AMF and +PA Low AMF) showed suppression of *PI-II* gene expression when compared to gene expression levels in plants with a high level of AM fungal root colonization (−PA High AMF and +PA High AMF). The mean gene expression levels in non-mycorrhizal plants (−PA Control and +PA Control) were similar to those in the other treatments (Figure 7).

## 3. Discussion

Root-associated beneficial microbes are known to prime host defenses by enhancing the expression of plant defense genes at the onset of insect and pathogen attack, thereby triggering induced systemic resistance (ISR). Nevertheless, this priming response is induced by stimuli from distinct microbial species or strains; ISR can be triggered by the JA/ET- or SA-signaling pathways, activating downstream defense genes [46]. In the case of MIR against pathogens or insects, the JA or SA pathways seem to predominate [20,21,29,47,48]. However, studies investigating the changes in plant-defense-gene expression in local tissues damaged by insects and systemic undamaged tissues in plants forming symbioses with AM fungi belowground are lacking. To date, a handful of studies involving aphid–plant–AM fungi interactions have used more than one level of AM fungal root colonization [27,29,30,43]. In the present study, a system involving potato aphids (*M. euphorbiae*), potato (*S. tuberosum* cv. Désirée), and the generalist AM fungus *R. intraradices* was established to (a) examine the indirect effect of AM fungal colonization of roots on potato aphid-potato interactions, (b) measure plant gene expression and determine whether potato plants with low and high levels of AM fungal root colonization show altered levels of defense transcripts, and (c) investigate whether aphid-damaged leaves and/or systemic undamaged leaves exhibit priming following root colonization by AM fungi.

**Figure 8:**
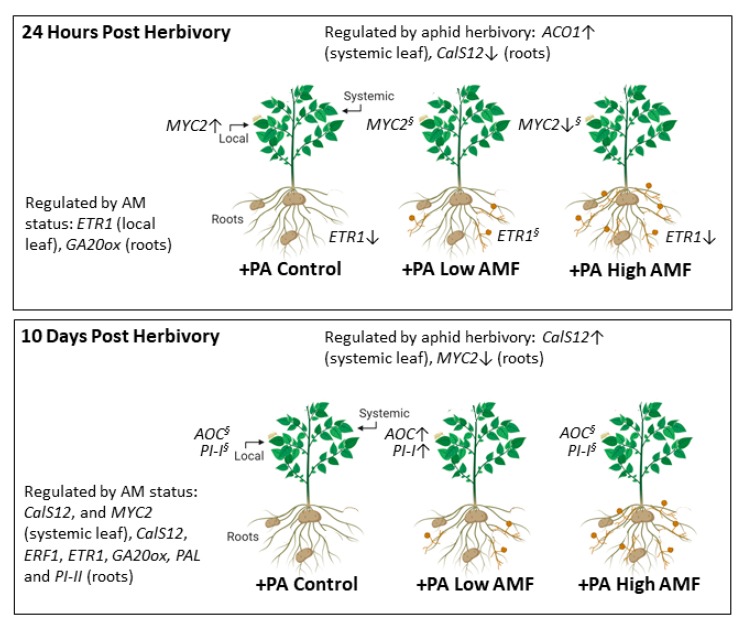
Summary of the effect of low and high level of AM fungal root colonization, 24 h and 10 days post-herbivory by potato aphids (*Macrosiphum euphorbiae*) on potato plants (*Solanum tuberosum*). For simplicity, only aphid-infested treatments such as potato-aphid-infested non-mycorrhizal plants (+PA Control), potato-aphid-infested plants with a low level of AM fungal root colonization (+PA Low AMF), and potato-aphid-infested plants with a high level of AM fungal root colonization (+PA High AMF) were included in this diagram. Changes in plant gene expression were mediated by aphid herbivory, AM status, or the interaction between potato aphid herbivory and AM fungal root colonization. Upregulation (↑) or downregulation (↓) of gene expression in local leaves, systemic leaves, and roots are shown. ^§^Gene expression level was not statistically different from the level in non-infested non-mycorrhizal plants (−PA Control). *ACO1 = AMINOCYCLOPROPANE-1-CARBOXYLATE OXIDASE 1; AOC = ALLENE OXIDE CYCLASE; CalS12 = CALLOSE SYNTHASE 12; ERF1 = ETHYLENE RESPONSE FACTOR 1; ETR1 = ETHYLENE RECEPTOR 1; GA20ox = GIBBERELIC ACID 20-OXIDASE; MYC2 = BASIC-HELIX-LOOP-HELIX TRANSCRIPTION FACTOR; PAL = PHENYLALANINE AMMONIA LYASE; PI-I = POTATO TYPE I PROTEASE INHIBITOR; PI-II = POTATO TYPE II PROTEASE INHIBITOR*. Data from Figure 2, Figure 3, Figure 4, Figure 5, Figure 6 and Figure 7 were used to create this diagram with BioRender.

The results indicate that ET (*ETR1*) and JA (*MYC2*) signaling may be involved in early regulation of aphid–potato–AM fungus interactions (Figure 2). When *ETR1* and *MYC2* gene expression levels were compared among aphid-infested plants, it was found that *ETR1* was downregulated in roots of aphid-infested non-mycorrhizal plants (+PA Control) and plants with a high level of AM fungal root colonization (+PA High AMF), compared to aphid-infested plants with a low level of AM fungal root colonization (+PA Low AMF) (Figure 8). On the contrary, *MYC2* gene expression was upregulated in local leaves of potato-aphid-infested non-mycorrhizal plants (+PA Control), as compared to potato-aphid-infested plants with a high level of AM fungal root colonization (+PA High AMF), indicating that JA signaling is suppressed in highly colonized plants (Figure 2 and Figure 8). In Arabidopsis, ET is perceived by five receptors, Ethylene Receptor (ETR) 1/ETR2, Ethylene Response Sensor (ERS) 1/ERS2, and Ethylene Insensitive (EIN) 4 that negatively regulate ET signaling [49,50,51]. The role of ET in regulating plant–aphid interactions has been examined primarily in leaves. It was found that potato aphids’ feeding in both resistant and susceptible tomato plants resulted in increased transcript levels of ET biosynthesis genes and ET production in leaflets [52]. The impairment of ET signaling or biosynthesis did not compromise *Mi-1*-mediated aphid resistance in tomato; however, it decreased susceptibility to the potato aphid in a compatible host [52]. ET signaling pathway genes (e.g., *ERT2*) and downstream response genes were strongly induced in *Vat*-aphid-resistant melon (*Cucumis melo*) after *Aphis gossypii* herbivory, implying that ET plays a role in *Vat*-mediated host-plant resistance [53]. Determining the role of ET signaling in the regulation of aphid–plant interactions belowground presents a promising avenue of future study.

The *AOC* and *PI-I* gene expression data at 10 days post-herbivory (Figure 5) supported our third hypothesis, which stated that potato-aphid-infested plants with a low level of AM fungal root colonization would show higher levels of defense-related transcripts in local leaves when compared with the levels in potato-aphid-infested non-mycorrhizal plants and plants with a high level of AM fungal root colonization. When *AOC* and *PI-I* gene expression levels in local leaves were compared among aphid-infested plants, it was found that gene expression was upregulated exclusively in aphid-infested plants with a low level of AM fungal root colonization (+PA Low AMF) (Figure 5 and Figure 8). Some studies have shown that these genes play a role in resistance against certain pests. In tomato plants, a strong induction of *LOXD*, *AOC*, *PI-I*, and *PI-II* genes in leaves started at 6 h post-feeding, increasing incrementally at 48 h post-feeding by the caterpillar *Helicoverpa arimigera* on mycorrhizal plants relative to non-mycorrhizal plants [21]. In this study, MIR was supported by the upregulation of JA-biosynthesis transcripts, and insect performance on JA overexpressing tomato plants, and both the *spr2* (JA deficient) and *jai1* (JA perception deficient) tomato mutants [21]. Overexpression of *AOC* in rice increased resistance to the piercing–sucking insect the brown planthopper (*Nilaparvata lugens*) by reducing insect feeding activity and survival rate [54]. However, five and eight days of feeding by the cabbage looper (*Trichoplusia ni*) on mycorrhizal potato did not change *AOC* gene expression in shoots or roots [48]. Knockdown or overexpression of *MtAOC1* in *M. truncatula* roots did not affect locally occurring bioprotection against the oomycete *Aphanomyces euteiches*, which is the causal agent of root rot in legumes [55]. On the other hand, strong induction of the *PI-I* gene in leaves of mycorrhizal plants after caterpillar herbivory has been reported in potato [48] and tomato plants [21]. Interestingly, upregulation of defenses in leaves such as PIs and chitinases has also been reported in ectomycorrhizal poplar (*Populus x canescens* (Aiton)) damaged by the poplar leaf beetle (*Chrysomela populi* L.) [56]. It was shown recently that co-expression of the barley serine and cysteine PIs in tomato plants resulted in increased host resistance to the South American tomato pinworm *Tuta absoluta* (Meyrick) [57]. In this study, gene expression patterns were distinguished by measuring transcript levels in local and systemic leaves, indicating that priming by AM fungal colonization of roots may be more targeted to local tissues attacked by aphids. Research with a focus on aphid–plant interactions has shown both systemic and local plant chemical induction after aphid feeding [58]. In pepper (*Capsicum annuum* L.), a transient systemic accumulation of JA and JA-isoleucine was observed in response to aphid (*M. persicae*) feeding at specific times post-infestation (8 and 48 h), whereas SA accumulation was the only local response at 96 h post-infestation [59]. In addition, primary aphid infestation increased plant resistance to a secondary aphid infestation at a systemic level in *M. truncatula* [60], but only in local tissue in *Arabidopsis* [61] and potato [62]. It would be interesting to investigate further if priming by AM fungal colonization of roots occurs in local and/or systemic tissue after a secondary aphid infestation.

The data also indicate that both local and systemic changes in plant gene expression aboveground and belowground appeared to be regulated primarily by AM status whether plants were damaged or not by potato aphids (Figure 8). In local leaves, *ETR1* gene expression differed among treatments (Control, Low AMF, and High AMF) at 24 h post-aphid-herbivory (Figure 4). *ETR1* gene expression increased in local leaves of plants with a low level of AM fungal root colonization compared to plants with a high level of AM fungal root colonization (Figure 4). The role of ET in the AM symbiosis has been studied by using tomato mutants that overproduce ET (*epi*) and fail to produce ET during fruit ripening (*rin*) [63]. It was found that *epi* plants had reduced AM-fungus-root-colonization intensity, and this was associated with a temporary increase in *ETR6* root transcript levels. On the other hand, AM fungal colonization parameters were positively affected in *rin* plants, indicating that the regulation of AM symbiosis is mediated by the RIN pathway in tomato plants [63]. Moreover, a modest induction of an ET biosynthesis gene (*ACO*) was observed in shoots of mycorrhizal *M. truncatula* plants [64]. ET may also be involved in regulating nodulation in soybean (*Glycine max*), as it was found that most soybean ET receptor genes (e.g., *GmERS1a*, *GmERS1b*, *GmEIN4c*, *GmETR1a*, *GmEIN4d*, and *GmERS2*) were responsive to root rhizobial infection by *Bradyrhizobium japonicum* [65]. At 10 days post-aphid-feeding, *CalS12* and *MYC2* gene expression in systemic leaves was reduced in plants with a high level of AM fungal root colonization (−PA High AMF and +PA High AMF) compared to the levels in non-mycorrhizal plants (−PA Control and +PA Control) and potato-aphid-infested plants with a low level of AM fungal root colonization (−PA Low AMF and +PA Low AMF), suggesting the suppression of systemic defenses (Figure 7). Callose is a branched polysaccharide that is synthesized to seal wounds in the phloem sieve elements [66]. When an aphid mouthpart pierces the phloem sieve element, the puncture in the plasma membrane is sealed, to avoid loss of phloem sap [67]. Plants are able to repair these wounds by depositing callose and proteins, but aphids modify these responses for their own benefit [68]. The *CalS12* gene, a homolog of *CalS12-like* from tomato that is involved in callose formation during pathogen infection [31], was downregulated in plants with a high level of AM fungal root colonization, but given that callose deposition was not measured in the present study, it is not possible to ascertain that transcript abundance of *CalS12* was correlated with callose formation [69]. The basic helix–loop–helix transcription factor MYC2 is known as a master regulator of JA signaling pathway (reviewed in [70]). MYC2 modulates the expression of early JA-response genes that include many transcription factors that regulate specific branches of the JA signaling pathway [70]. The Arabidopsis *myc2* mutant shows compromised rhizobacteria-ISR to the bacterial pathogen *Pseudomonas syringae* pv. tomato (*Pst*) DC3000 and the fungal pathogen *Hyaloperonospora parasitica* [71]. In addition, the colonization of *Arabidopsis* roots by rhizobacteria primes many JA-responsive genes in shoots, suggesting that MYC2 regulates this priming response [71]. *MYC2* transcripts also increased in plants simultaneously attacked by aphids at a high density and caterpillars [71]. It was concluded that reduced gene expression of SA transcription factor *WRKY70* led to higher *MYC2* gene expression through SA–JA cross-talk [71]. In this study, the aboveground suppression of *MYC2* gene expression in plants with a high level of AM fungal colonization of roots may indicate that these plants have a compromised induced systemic defense response.

It is noteworthy that several genes (*CalS12*, *ERF1*, *ETR1*, *GA20ox*, *PAL*, and *PI-II*) were differentially expressed in roots at 10 days post-aphid-herbivory (Figure 7). *GA20ox*, a gene involved in the synthesis of bioactive gibberellins, has been reported to be induced in roots of mycorrhizal *M. truncatula* plants without aphids [72] and plants with aphids feeding on the shoots [29]. *GA20ox* root transcript levels increased in plants with a high level of AM fungal colonization of roots (−PA High AMF and +PA High AMF) compared to non-mycorrhizal plants (−PA Control and +PA Control) at 24 h and at 10 days post-aphid-herbivory (Figure 4 and Figure 7). However, the reported downregulation of *GA20ox* transcripts by pea aphids feeding on shoots and roots of non-mycorrhizal and mycorrhizal *M. truncatula* plants [29] was not observed in the present study, indicating that regulation of this gene is either insect-density-dependent or differs between plant families. In the pea aphid–*M. truncatula*–AM fungus study, the aphid density was higher, and insects fed on all aboveground tissue [29], which could explain the differences in *GA20ox* gene expression. These defense-gene-expression data provide intriguing information regarding aphid success on highly colonized plants that was reported in other systems [27,29,34,43].

Likewise, local and systemic changes in plant gene expression aboveground and belowground were triggered by potato aphid herbivory, whether plants were colonized or not by AM fungi (Figure 3, Figure 6 and Figure 8). Interestingly, 24 h and 10 days of herbivory by potato aphids resulted in upregulation of *ACO1* and *CalS12* transcripts in systemic leaves, respectively. The feeding activity of green peach aphids (*M. persicae*) increased while feeding on potato leaves that had been previously infested by green peach aphids (conspecific) or potato aphids (heterospecific). On the contrary, the feeding activity was inhibited on non-infested leaves of aphid-infested plants, supporting a mechanism of induced resistance [62]. In the present study, *CalS12* and *MYC2* gene expression in roots was suppressed by potato aphid herbivory at 24 h and at 10 days (Figure 3 and Figure 6). Conversely, induction of *MYC2* root transcripts was observed after a seven-day herbivory by pea aphids on *M. truncatula* plants that had a high level of AM fungal root colonization, compared to highly colonized mycorrhizal plants without aphids, where *MYC2* transcripts were repressed [29]. It is possible that potato aphids’ feeding on potato results in reprogramming of plant defense responses that are more targeted to aboveground plant tissues that are under attack [62,73].

In the present study, aphid colony weight and aphid number per colony did not change after feeding on non-mycorrhizal and mycorrhizal potato for 24 h and 10 days (Figure 1). The data did not support the hypotheses that (1) aphids would show decreased aphid fitness after feeding for 10 days on potato plants with a low level of AM fungal root colonization, or that (2) aphids would show improved aphid fitness after feeding for 10 days on potato plants with a high level of AM fungal root colonization. A possible reason for not observing a change in aphid fitness (abundance and weight) in this study could be attributed to using adult aphids (>10 days old), and this should be considered in future experiments. It has been reported that green peach aphids (*M. persicae*) that fed on young *P. lanceolata* plants in an early stage of the AM symbiosis (20 days postinoculation, dpi; 10% RLC) had a lower relative growth rate (RGR) compared to those on young non-mycorrhizal plants, while aphids that fed on old *P. lanceolata* plants with a well-established AM symbiosis (62 dpi; 80% RLC) had a higher RGR compared to those on old non-mycorrhizal plants [43]. The body mass of six-day-old *M. persicae* nymphs (F1 generation) was lower on old plants with a well-established AM symbiosis compared to old non-mycorrhizal plants, but because of the higher RGR on mycorrhizal plants, 10-day-old aphids reached a similar body mass on non-mycorrhizal and mycorrhizal plants [43]. Potato aphid survival improved on tomato plants colonized by the AM fungus *R. intraradices* during exposure to water deficit and well-watered conditions. Interestingly, indirect defenses (e.g., methyl salicylate) increased in mycorrhizal tomato plants, resulting in attraction of the aphid parasitoid *Aphidius ervi* Haliday [74]. It is possible that a similar type of indirect defense response occurs in this potato aphid–potato–*R. intraradices* system. In the present study, plant age was the same, but the level of AM fungal root colonization varied. It is also possible that potato aphids may have compensated for suboptimal food, as was reported for *M. persicae* [43]. Future studies using this potato aphid–potato–AM fungi system should consider using younger aphids (<10 days old), measure additional parameters of aphid fitness (e.g., RGR) at different time-points post-herbivory, and test aphid density [75,76] to gain a more complete picture of the effects of AM-fungi-modulated plant–insect herbivore interactions.

## 4. Materials and Methods

### 4.1. Plant Growth Conditions

Potato (*Solanum tuberosum* cv Désirée) was in vitro propagated by using nodal cuttings that were maintained in tissue culture glass tubes containing Murashige and Skoog (MS) medium supplemented with sucrose (20 g L^−1^) and phytagel (3 g L^−1^), at pH = 5.7 [77]. Plantlets were grown in an incubator at 22 °C and 16:8 (L:D) h cycle for 6 weeks prior to transplanting into plastic pots (6.4 cm H × 6.4 cm W × 8.9 cm D) filled with autoclaved 9:1 (sand:topsoil) substrate. The substrates utilized for the experiment were based on previous results [29] and were composed of a mixture of eight parts mason sand and one part topsoil (Pioneer Sand Company, Windsor, CO, USA). Plants were covered with a plastic humidity dome (54.6 cm H × 28 cm W × 17.8 cm D) for 1 week and were grown under laboratory conditions on wire shelving (152 × 60 cm), under a canopy of four fluorescent bulbs (200–230 µmol m^−2^ s^−1^) per shelf, using a 16-hour photoperiod at 23–24 °C. Topsoil was sieved (USA Standard sieve No. 16 and 8) to ensure uniformity before autoclaving for three cycles (121 °C, 20 psi, 60 min each cycle). Mason sand was rinsed with deionized water multiple times and was autoclaved for one cycle (121 °C, 20 psi, 60 min). Nutrient analysis of the mixed autoclaved sand–topsoil substrate was conducted at Weld Laboratories in Greeley, CO, and showed a pH = 7.18, organic matter of 0.1%, nitrogen level of 3.4 ppm, phosphorus level of 4.0 ppm, and potassium level of 20.0 ppm. After transplant to the sand–topsoil substrate, plants received 35 mL of ½ strength Hoagland’s solution twice per week, modified from standard Hoagland’s solution with 100 µM P (reduced) at pH 6.1. On all days without fertilizer, plants were watered consistently with tap water. The age of the potato plants calculated from the day of in vitro propagation ranged from 64 to 73 d at harvest.

### 4.2. Root Inoculation with the AM Fungus Rhizophagus Intraradices

After a 1 week acclimation period of plants to the sand–topsoil substrate, a group of potato plants were inoculated with a 1:10 dilution of the soil inoculum containing *Rhizophagus intraradices* UT118, IA506, and CO204, while another group of plants received a 1:25 dilution of the *R. intraradices* inoculum, to obtain two levels of AM fungus root colonization, “High AMF” and “Low AMF”, respectively. The “High AMF” inoculum was prepared by mixing one part of *R. intraradices* inoculum with eight parts of autoclaved sand–topsoil substrate, whereas the “Low AMF” inoculum was prepared by mixing one part of soil inoculum with 24 parts of autoclaved sand–topsoil substrate. Non-mycorrhizal “Control” plants received a 1:25 dilution of inoculum that contained root exudates devoid of *R. intraradices*. Seventeen plants were inoculated with each level of AM fungus inoculum (each plant was considered a replicate), which included three extra inoculated plants per inoculum to assess the root colonization levels prior to adding aphids. Both *R. intraradices* and non-mycorrhizal control inocula were purchased from Dr. Joe Morton (International Culture Collection of (Vesicular) Arbuscular Mycorrhizal Fungi, INVAM). At 1 week postinoculation, plants were watered as needed and fertilized twice per week with 35 mL ½ strength modified Hoagland’s solution with 100 μM P (reduced) pH 6.1 [78]. Plants were grown for 5 weeks, until AM fungus root colonization levels of extra plants reached at least 20% root length colonized (RLC) for “Low AMF” and at least 60% RLC for “High AMF”. To assess AM fungal colonization level, roots were cleared in 10% (*w*/*v*) KOH at 85 °C for 4–6 h, rinsed in deionized water, incubated in 5% (*v*/*v*) glacial acetic acid at room temperature for 10 min, incubated in a 5% (*v*/*v*) Shaeffer^®^ black Skrip^®^ ink (A.T. Cross Company, Providence, RI) staining solution prepared in 5% (*v*/*v*) glacial acetic acid, and excess ink was rinsed out in deionized water [79]. Root colonization by *R. intraradices* was quantified via the magnified intersections method [80], using a SZX10 stereo microscope (Olympus America Inc., Center Valley, PA, USA). 

### 4.3. Shoot Infestation with Potato Aphids

Potato aphids (PAs, *M. euphorbiae*) isolate WU11 were kindly provided by Dr. Fiona Goggin (University of Arkansas) and were reared on broad bean (*Vicia faba*) in BugDorm-2120 insect-rearing tents, kept at 23–24 °C, under a 16:8 (L:D) h cycle, at light quanta of 200–230 µmol m^−2^ s^−1^, and fertilized with 35 mL Miracle-Gro water-soluble all-purpose plant food (Scotts, Marysville, OH, USA) twice per week. To ensure insects of similar age, aphids were synchronized two weeks prior to placement on experimental plants by removal and transplant of 1-day-old nymphs onto non-infested broad bean plants. Aphids used in the experiment were between 10 to 14 days old. When mycorrhizal plants reached the target levels of AM fungal colonization of roots (≥20% RLC for “Low AMF”; ≥60% RLC for “High AMF”), all plants were transferred to a greenhouse under a 16:8 (L:D) h cycle at 24 °C. Plants were placed into BugDorm-2120 insect-rearing tents (MegaView Science Co., Ltd., Taichung, Taiwan), in groups of seven biological replicates per tent per treatment. The study included six experimental treatments as follows: (1) non-infested non-mycorrhizal plants (−PA Control), (2) potato-aphid-infested non-mycorrhizal plants (+PA Control), (3) non-infested plants with a low level of AM fungal root colonization (−PA Low AMF), (4) potato-aphid-infested plants with a low level of AM fungal root colonization (+PA Low AMF), (5) non-infested plants with a high level of AM fungal root colonization (−PA High AMF), and (6) potato-aphid-infested plants with a high level of AM fungal root colonization (+PA High AMF). Plants were allowed a 1-week acclimation period to greenhouse conditions and tents prior to the addition of aphids. For all potato-aphid-infested treatments (+PA), four apterous adults were placed on the terminal leaflet of the 4th fully expanded leaf, counting from the shoot apex, with the leaf enclosed in an organza drawstring bag (10 × 15 cm) and sealed at the petiole. Each aphid-infested leaf per plant represents a “Local leaf”, while the 3rd fully expanded leaf represents a “Systemic leaf”. Similarly, the 3rd fully expanded leaf, counting from the shoot apex, was bagged devoid of aphids. The 3rd and 4th fully expanded leaves on all non-infested treatments (−PA) were also bagged.

### 4.4. Investigating the Impact of AM Fungus Root Colonization on Potato Aphid Fitness

The indirect effect of the AM fungus *R. intraradices* on potato aphid–potato interactions was examined based on the insect’s ability to grow, and reproduce on mycorrhizal plants. At 24 h and 10 days after aphid infestation, the total number of live aphids (adults and nymphs) was recorded in each “Local” leaf from mycorrhizal (Low AMF and High AMF) and non-mycorrhizal (Control) plants. Potato aphids were carefully removed from each “Local” leaf with a paintbrush and were frozen at 20 °C overnight inside 40 mm culture dishes. Non-infested (−PA) “Local” leaves were also brushed with a paintbrush. All live aphids that were present in a colony of each plant were counted and weighed by using an MX5 microbalance (Mettler Toledo, Columbus, OH, USA). Root subsamples from mycorrhizal and non-mycorrhizal plants were collected, cleared in 10% (*w*/*v*) KOH at 85 °C for 5 h, rinsed in deionized water, incubated in 5% (*v*/*v*) glacial acetic acid at room temperature for 10 min, incubated in a 5% (*v*/*v*) Shaeffer^®^ black Skrip^®^ ink staining solution prepared in 5% (*v*/*v*) glacial acetic acid, and excess ink was rinsed out in deionized water. *R. intraradices* root colonization was quantified via the magnified gridline-intersection method [80]. Roots from non-mycorrhizal plants did not show *R. intraradices* staining. Each “Local” and “Systemic” leaf was frozen separately in liquid nitrogen, without recording its weight. The remaining fresh shoot and root tissues were weighed immediately before freezing in liquid nitrogen, and all samples were stored at −80 °C. At harvest, plants were 36- and 45-days postinoculation.

### 4.5. RNA Isolation and cDNA Synthesis

The three biological replicates closest to the mean shoot and root fresh weights, %RLC, and aphid population from each treatment from each aphid feeding period were selected for gene expression analysis. Potato “Local” and “Systemic” leaves and root samples were ground in liquid nitrogen using a mortar and pestle. RNA was extracted by using the RNeasy plant mini kit according to the manufacturer’s instructions (Qiagen Inc., Germantown, MD, USA). Subsequently, RNA was treated with 5 µL of Turbo^TM^ DNase (2 Units µL^−1^) (Thermo Fisher Scientific, Waltham, MA, USA) and 10 µL of 10X reaction buffer to a total volume of 100 µL, and samples were incubated at 37 °C for 40 min. DNAse-treated RNA samples were subsequently purified using the RNeasy MinElute Cleanup kit (Qiagen Inc.). For cDNA synthesis, 2 µg of total RNA was mixed with 2 µL dNTPs (10 mM each) and 2 µL anchored oligo dT_22_ (500 ng µL^−1^) and incubated at 65 °C for 5 min. Next, 8 µL of SuperScript^®^ IV buffer, 2.4 µL of Nuclease-free water, 1 µL of DTT (100 mM), 0.6 µL of SuperScript^®^ IV (Thermo Fisher Scientific), and 1 µL of RNase OUT^TM^ (Thermo Fisher Scientific) were added to each sample (total volume 40 µL), and samples were incubated at 50 °C for 10 min, and 80 °C for 10 min. The cDNA quality was assessed by reverse-transcription polymerase chain reaction (RT–PCR) (27 cycles of 95 °C for 30 sec, 59 °C for 30 sec, and 72 °C for 30 sec), using the reference gene *ELONGATION FACTOR 1 α* (*EF1-α*) [48,81]. Products were visualized on a 0.5X TAE 2% (*w*/*v*) agarose gel. Only cDNA replicates that showed similar expression levels of *EF1-α* were used for RT–quantitative real-time PCR (RT–qPCR).

### 4.6. Determining Changes in Plant Gene Expression during Aphid–Plant–AM Fungus Interactions

Plant gene expression was measured to do the following: (a) determine whether mycorrhizal potato plants with low and high levels of AM fungal root colonization show altered levels of defense-related transcripts; and (b) investigate whether aphid-damaged leaves and/or systemic undamaged leaves exhibit priming following AM fungal root colonization. First, potato sequences were obtained via the Basic Local Alignment Search Tool (BLAST) from the National Center for Biotechnology Information (NCBI) and SPUD database (potato.plantbiology.msu.edu/). Query sequences from either tomato, tobacco, Arabidopsis, or *Medicago truncatula* (based on gene availability) were used for BLAST searches. Oligonucleotides were designed by using Primer 3 (http://bioinfo.ut.ee/primer3-0.4.0/primer3/) or Primer-BLAST (https://www.ncbi.nlm.nih.gov/tools/primer-blast/). Genes of interest included those involved in JA biosynthesis [*ALLENE OXIDE CYCLASE* (*AOC*)] [48,82], JA signaling [transcription factor *MYC2*] [29], ethylene (ET) biosynthesis and signaling [*1-AMINOCYCLOPROPANE-1-CARBOXYLATE OXIDASE* (*ACO1*), *ETHYLENE RESPONSE FACTOR 1* (*ERF1*) and *ETHYLENE RECEPTOR 1* (*ETR1*)] [29,83,84], GA pathway [*GA 20-OXIDASE* (*GA20ox*)] [29,72], callose formation during pathogen infection [*CALLOSE SYNTHASE-LIKE 12* (*CalS12*)] [31], phenylpropanoid biosynthesis [*PHENYLALANINE AMMONIA LYASE* (*PAL*)] [48,85], and genes associated with plant defenses to insects such as *POTATO TYPE I* and *II PROTEASE INHIBITORS* (*PI-I* and *PI-II*) [21,48].

RT–qPCR efficiency was determined by using a 10-fold serial dilution of cDNAs (undiluted, 1:10, 1:100, 1:1000, and 1:10,000) with each oligonucleotide pair, and the R^2^ and efficiency values are reported in Table S3. Each RT–qPCR reaction consisted of 1 µL of cDNA template (1:3), 5 µL of Power SYBR^®^ Green Master Mix (Thermo Fisher Scientific), 2 µL of autoclaved Milli-Q^®^ water, and 1 µL each of 3 µM forward and reverse primers. Each 384-well plate was run on a C1000^®^ Touch Thermal Cycler (Bio-Rad, Hercules, CA, USA), with two technical replicates included in every run. The thermal profile consisted of an incubation at 95 °C for 10 min, followed by 40 cycles at 95 °C for 15 sec and annealing/extension at 55–60 °C for 1 min, ending with melt curve analysis (65–95 °C incrementally increasing by 5 °C). The oligonucleotide sequences and annealing temperatures used for RT–qPCR are reported in Table S3. The 2^−ΔCq^ method was used to calculate the relative expression of 10 gene targets [86], where each gene was calibrated to the reference gene *EF1-α* [48,81,87].

### 4.7. Statistical Analyses

Raw data were tested for normality by using the Shapiro–Wilk and Anderson–Darling tests. Data that were not normally distributed were subjected to Box-Cox transformation prior to using parametric tests. Two-factor analyses of variance (ANOVA) were used to analyze shoot fresh weight, root fresh weight, and relative gene expression. One-factor ANOVA was used when the interaction term (Potato aphid herbivory and AM fungal root colonization, PA*AMF) was significant or when the main effect AM fungal root colonization (AMF) was significant. The Tukey–Kramer test was used when *p* < 0.05 in the one-factor ANOVA. Student’s *t*-tests were used when the main effect potato aphid herbivory (PA) was significant. In addition, one-factor ANOVA was used for %RLC, aphid colony weight, and aphid number. All statistical analyses were conducted by using SAS 9.4 for Windows (SAS Institute Inc., Cary, NC, USA).

## 5. Conclusions

The present study developed a system to investigate potato-aphid–potato-AM-fungus interactions at the molecular level. Few studies involving this type of interaction used more than one level of AM fungal colonization of roots [27,29,30,43]. In most cases, aphid responses after feeding on non-mycorrhizal plants were compared to plants that were either low colonized or highly colonized by AM fungi. This study expands the knowledge on plant responses to aphid herbivory when plants of the same age have distinct levels of AM fungal colonization of roots. This research examined the changes in plant gene expression, focusing on local leaves infested with aphids, systemic non-infested leaves, and roots during a tripartite interaction, an aspect that has been less considered so far. Plant gene expression of ten defense-related genes at two time-points post-aphid-herbivory (24 h and 10 days) in three tissue types was assessed. The findings indicate that ET and JA signaling may be involved in early regulation of aphid–potato interactions. At 24 h post-herbivory, *ETR1* gene expression was repressed in roots of potato-aphid-infested non-mycorrhizal plants and potato-aphid-infested plants with a high level of AM fungal root colonization, but not on aphid-infested plants with a low level of AM fungal root colonization. *MYC2* gene expression was upregulated in local leaves of potato-aphid-infested non-mycorrhizal plants compared to plants with a high level of AM fungal root colonization. At 10 days post-herbivory, two defense genes, *AOC* and *PI-I,* were upregulated exclusively in local leaves of aphid-infested plants with a low level of AM fungal root colonization, indicating that plant defenses are targeted to plant tissues under attack. The data also indicate that both local and systemic changes in plant gene expression aboveground and belowground appeared to be regulated by (a) AM status, whether plants were infested or not by potato aphids, and (b) potato aphid herbivory, whether plants were colonized or not by AM fungi. Even though the parameters of insect fitness measured in this study did not change, the gene-expression data provide insights on potato responses to beneficial root-associated microbes and foliar aphid herbivory and help with the design of future experiments aimed at better understanding the molecular mechanisms involved in modulating tripartite interactions.

## Supplementary Materials

The following are available online at https://www.mdpi.com/2223-7747/9/1/82/s1. Figure S1: Percentage of potato (*Solanum tuberosum*) root length colonized by the AM fungus *Rhizophagus intraradices* following (a) 24 h and (b) 10 days of potato aphid (*Macrosiphum euphorbiae*) herbivory. Values represent the mean of seven biological replicates ± SEM per treatment for the 24 h and 10 days feeding time-points. Different letters indicate significant differences among treatments based on the Tukey–Kramer test (*p* ≤ 0.05). PA = potato aphid; Low AMF = plants with a low level of AM fungal root colonization; High AMF = plants with a high level of AM fungal root colonization. Figure S2: Effect of distinct levels of AM fungal root colonization on (a) shoot and (b) root growth of potato plants (*Solanum tuberosum*). Fresh weight of potato plants at 24 h after aphid (*Macrosiphum euphorbiae*) herbivory. Values represent the mean of seven biological replicates ± SEM. A *p* ≥ 0.05 indicates no statistical significance based on ANOVA. Non-infested non-mycorrhizal plants (−PA Control); potato-aphid-infested non-mycorrhizal plants (+PA Control); non-infested plants with a low level of AM fungal root colonization (−PA Low AMF); potato-aphid-infested plants with a low level of AM fungal root colonization (+PA Low AMF); non-infested plants with a high level of AM fungal root colonization (−PA High AMF); and potato-aphid-infested plants with a high level of AM fungal root colonization (+PA High AMF). Figure S3: Effect of distinct levels of AM fungal root colonization on (a) shoot and (b) root growth of potato plants (*Solanum tuberosum*). Fresh weight of potato plants at 10 days after aphid (*Macrosiphum euphorbiae*) herbivory. Values represent the mean of seven biological replicates ± SEM. A *p* ≥ 0.05 indicates no statistical significance based on ANOVA. Non-infested non-mycorrhizal plants (−PA Control); potato-aphid-infested non-mycorrhizal plants (+PA Control); non-infested plants with a low level of AM fungal colonization of roots (−PA Low AMF); potato-aphid-infested plants with a low level of AM fungal root colonization (+PA Low AMF); non-infested plants with a high level of AM fungal root colonization (−PA High AMF); and potato-aphid-infested plants with a high level of AM fungal root colonization (+PA High AMF). Table S1: Two-factor ANOVA of relative gene expression by tissue type at 24 h post-aphid-herbivory. Table S2: Two-factor ANOVA of relative gene expression by tissue type at 10 days post-aphid-herbivory. Table S3: Information about oligonucleotides and annealing temperatures used for RT–qPCR.
