# Supplementary material for: Assessment of Local and Systemic Changes in Plant Gene Expression and Aphid Responses during Potato Interactions with Arbuscular Mycorrhizal Fungi and Potato Aphids
Source: Plants (Basel). 2020 Jan 9;9(1):82. doi: 10.3390/plants9010082 (PMC7020417; doi:10.3390/plants9010082)
Supplement: Supplementary file 1 [file plants-09-00082-s001.zip › Revised Suppl Files/Table S1.docx]

**Table S1.** Two-Factor ANOVA of relative gene expression by tissue type at 24 h post aphid herbivory.

| **Gene** | **Tissue Type** | **PA**  ***P* value** | **AMF**  ***P* value** | **PA*AMF**  ***P* value** |
| --- | --- | --- | --- | --- |
| *ACO1* | Local leaf | 0.5382 | 0.2866 | 0.8596 |
|  | Systemic leaf | 0.0395* | 0.3360 | 0.8491 |
|  | Roots | 0.1980 | 0.6371 | 0.6071 |
| *AOC* | Local leaf | 0.2158 | 0.7236 | 0.5852 |
|  | Systemic leaf | 0.5451 | 0.2864 | 0.7553 |
|  | Roots | 0.2456 | 0.6443 | 0.8243 |
| *CalS12* | Local leaf | 0.9264 | 0.4411 | 0.3443 |
|  | Systemic leaf | 0.5984 | 0.3732 | 0.9952 |
|  | Roots | 0.0267* | 0.1447 | 0.2117 |
| *ERF1* | Local leaf | 0.2651 | 0.5302 | 0.4779 |
|  | Systemic leaf | 0.2320 | 0.2174 | 0.3690 |
|  | Roots | 0.9793 | 0.4100 | 0.6113 |
| *ETR1* | Local leaf | 0.1664 | 0.0306* | 0.2450 |
|  | Systemic leaf | 0.7672 | 0.2278 | 0.2341 |
|  | Roots | 0.8971 | 0.0147* | <0.0001* |
| *GA20ox* | Local leaf | 0.7304 | 0.3963 | 0.0889 |
|  | Systemic leaf | 0.8233 | 0.9452 | 0.1297 |
|  | Roots | 0.1402 | 0.0030* | 0.4114 |
| *MYC2* | Local leaf | 0.0004* | 0.0368* | 0.0398* |
|  | Systemic leaf | 0.3845 | 0.4732 | 0.5934 |
|  | Roots | 0.2740 | 0.3791 | 0.3795 |
| *PAL* | Local leaf | 0.8995 | 0.7647 | 0.2337 |
|  | Systemic leaf | 0.8959 | 0.9290 | 0.2426 |
|  | Roots | 0.9018 | 0.2167 | 0.2758 |
| *PI-I* | Local leaf | 0.3089 | 0.2778 | 0.1557 |
|  | Systemic leaf | 0.1239 | 0.0981 | 0.1025 |
|  | Roots | 0.9378 | 0.3742 | 0.0597 |
| *PI-II* | Local leaf | 0.1929 | 0.1424 | 0.0565 |
|  | Systemic leaf | 0.7579 | 0.1741 | 0.8923 |
|  | Roots | 0.0789 | 0.1565 | 0.0859 |

PA = potato aphid; AMF = arbuscular mycorrhizal fungi

**P* ≤ 0.05 indicates statistical difference
