# Supplementary material for: Assessment of Local and Systemic Changes in Plant Gene Expression and Aphid Responses during Potato Interactions with Arbuscular Mycorrhizal Fungi and Potato Aphids
Source: Plants (Basel). 2020 Jan 9;9(1):82. doi: 10.3390/plants9010082 (PMC7020417; doi:10.3390/plants9010082)
Supplement: Supplementary file 1 [file plants-09-00082-s001.zip › Revised Suppl Files/Table S2.docx]

**Table S2.** Two-Factor ANOVA of relative gene expression by tissue type at 10 days post aphid herbivory.

| **Gene** | **Tissue Type** | **PA**  ***P* value** | **AMF**  ***P* value** | **PA*AMF**  ***P* value** |
| --- | --- | --- | --- | --- |
| *ACO1* | Local leaf | 0.2240 | 0.0806 | 0.6875 |
|  | Systemic leaf | 0.4595 | 0.5120 | 0.2115 |
|  | Roots | 0.4465 | 0.4120 | 0.1621 |
| *AOC* | Local leaf | 0.0835 | 0.0019* | 0.0008* |
|  | Systemic leaf | 0.3314 | 0.5479 | 0.5272 |
|  | Roots | 0.6142 | 0.1851 | 0.8326 |
| *CalS12* | Local leaf | 0.8654 | 0.0957 | 0.6549 |
|  | Systemic leaf | 0.0166* | 0.0017* | 0.2118 |
|  | Roots | 0.0850 | 0.0402* | 0.2667 |
| *ERF1* | Local leaf | 0.5215 | 0.5607 | 0.4070 |
|  | Systemic leaf | 0.3305 | 0.4245 | 0.4516 |
|  | Roots | 0.8104 | 0.0433* | 0.7473 |
| *ETR1* | Local leaf | 0.0676 | 0.2304 | 0.7018 |
|  | Systemic leaf | 0.3440 | 0.4256 | 0.4149 |
|  | Roots | 0.1155 | 0.0273* | 0.6008 |
| *GA20ox* | Local leaf | 0.8175 | 0.5896 | 0.3263 |
|  | Systemic leaf | 0.3445 | 0.4906 | 0.4397 |
|  | Roots | 0.1599 | 0.0010* | 0.3588 |
| *MYC2* | Local leaf | 0.0853 | 0.0690 | 0.4013 |
|  | Systemic leaf | 0.9058 | 0.0032* | 0.5771 |
|  | Roots | 0.0219* | 0.2331 | 0.0912 |
| *PAL* | Local leaf | 0.1176 | 0.8064 | 0.5147 |
|  | Systemic leaf | 0.2784 | 0.6608 | 0.5702 |
|  | Roots | 0.7745 | 0.0363* | 0.4034 |
| *PI-I* | Local leaf | 0.0625 | 0.0465* | 0.0455* |
|  | Systemic leaf | 0.3443 | 0.1827 | 0.3674 |
|  | Roots | 0.4534 | 0.4156 | 0.1041 |
| *PI-II* | Local leaf | 0.1057 | 0.5320 | 0.6109 |
|  | Systemic leaf | 0.8282 | 0.3463 | 0.7379 |
|  | Roots | 0.9101 | 0.0341* | 0.6261 |

PA = potato aphid; AMF = arbuscular mycorrhizal fungi

**P* ≤ 0.05 indicates statistical difference
