# Supplementary material for: Assessment of Local and Systemic Changes in Plant Gene Expression and Aphid Responses during Potato Interactions with Arbuscular Mycorrhizal Fungi and Potato Aphids
Source: Plants (Basel). 2020 Jan 9;9(1):82. doi: 10.3390/plants9010082 (PMC7020417; doi:10.3390/plants9010082)
Supplement: Supplementary file 1 [file plants-09-00082-s001.zip › Revised Suppl Files/Table S3.docx]

Table S3. Information about oligonucleotides and annealing temperatures used for RT-qPCR.

| Gene | Forward (F) and Reverse (R) Oligonucleotide Sequences (5' to 3') | Amplicon length (bp) | Annealing temperature (°C) | R^2^ | Primer Efficiency (%) | Plant species used to find potato sequence | Potato sequence ID |
| --- | --- | --- | --- | --- | --- | --- | --- |
| *ELONGATION FACTOR 1-α* (*EF1-α*) | F-GAGACCTTTGCTGAATACCCAC  R-TCACTTTGGCACCAGTTGG | 118 | 56.7 | 0.9970 | 99.4% | Potato [1] | AB061263.1 |
| *1-AMINOCYCLOPROPANE-1-CARBOXYLATE OXIDASE (ACO1)* | F-ACTTTGGTTGAAAAAGAGGCAGAG  R-AATTGGATCACTTTCCATTGCC | 147 | 55.0 | 0.9982 | 108.3% | Tomato [2] | PGSC0003DMT400041796 |
| *ALLENE OXIDE CYCLASE (AOC)* | F- AGTTGTTGTGTACGGCGGTT  R- GCACATCAACACCCCCACTT | 119 | 59.6 | 0.9983 | 99.6% | Tomato [3] | PGSC0003DMT400033027 |
| *CALLOSE SYNTHASE 12-LIKE (CalS12)* | F-GCCCAAGTGTTACGTCCCTT  R-AACCAGGCAACCAAGACAGT | 133 | 57.0 | 0.9955 | 93.1% | Tomato [4] | Sotub02g011920.1.1 |
| *ETHYLENE RESPONSE FACTOR 1 (ERF-1)* | F-CCTTCTCGCACAAGCTGACT  R-CCGACGCCTAACACCTCTAT | 112 | 57.0 | 0.9931 | 98.1% | Potato [5] | PGSC0003DMT400034873 |
| *ETHYLENE RECEPTOR 1 (ETR1)* | F-TCCTAAAACCTGTGTCAGTGGAT  R-GTTGCTGCACATTTTCCACCTA | 108 | 56.0 | 0.9921 | 94.3% | Arabidopsis | XM_006349934.2 |
| *GIBBERELLIC ACID 20-OXIDASE (GA20ox)* | F-AGGCGTACAGAAGAACCACTT  R-GCCATGTTCCTAAGGTGAGC | 110 | 56.0 | 0.9957 | 79.4% | Medicago [6] | PGSC0003DMT400036523 |
| *TRANSCRIPTION FACTOR MYC2 (MYC2)* | F-CCACAGTGAAAATGGGTAGCAG  R-TTCAAAGCCCTCGACGATTTCT | 115 | 56.0 | 0.9936 | 87.8% | Tomato | Sotub10g009150.1.1 |
| *PHENYLALANINE AMMONIA LYASE* (*PAL*) | F- CCTAGTAGACCACGCCTTGC  R- GGGTTTCCACTTTCCAACGC | 150 | 60.0 | 0.9903 | 101.0% | Medicago [7] | PGSC0003DMT400080548 |
| *POTATO TYPE I PROTEASE INHIBITOR* (*PI-I*) | F- CGTTGTAATCGAGTTCGTCTTGT  R- TGACATGTGGCTGCTTACTTCA | 103 | 56.7 | 0.9859 | 100.0% | Tomato [8] | PGSC0003DMT400031525 |
| *POTATO TYPE II PROTEASE INHIBITOR (PI-II)* | F- AATTGTTGTACCGCAGGAGAGG  R- CCAACTTGGTTATGCTGTACTGG | 99 | 59.1 | 0.9840 | 101.7% | Tomato [8] | PGSC0003DMT400039544 |

**References**

1. Dou, H.O.; Xv, K.P.; Meng, Q.W.; Li, G.; Yang, X.H. Potato plants ectopically expressing *Arabidopsis thaliana* CBF3 exhibit enhanced tolerance to high-temperature stress. *Plant Cell and Environment* **2015**, *38*, 61-72, doi:10.1111/pce.12366.

2. Nie, X.Z.; Singh, R.P.; Tai, G.C.C. Molecular characterization and expression analysis of 1-aminocyclopropane-1-carboxylate oxidase homologs from potato under abiotic and biotic stresses. *Genome* **2002**, *45*, 905-913, doi:10.1139/g02-062.

3. Lopez-Raez, J.A.; Verhage, A.; Fernandez, I.; Garcia, J.M.; Azcon-Aguilar, C.; Flors, V.; Pozo, M.J. Hormonal and transcriptional profiles highlight common and differential host responses to arbuscular mycorrhizal fungi and the regulation of the oxylipin pathway. *J. Exp. Bot.* **2010**, *61*, 2589-2601, doi:10.1093/jxb/erq089.

4. Adkar-Purushothama, C.R.; Brosseau, C.; Giguere, T.; Sano, T.; Moffett, P.; Perreault, J.P. Small RNA derived from the virulence modulating region of the potato spindle tuber viroid silences callose synthase genes of tomato plants. *Plant Cell* **2015**, *27*, 2178-2194, doi:10.1105/tpc.15.00523.

5. Wiesel, L.; Davis, J.L.; Milne, L.; Fernandez, V.R.; Herold, M.B.; Williams, J.M.; Morris, J.; Hedley, P.E.; Harrower, B.; Newton, A.C., et al. A transcriptional reference map of defence hormone responses in potato. *Sci. Rep.* **2015**, *5*, doi:10.1038/srep15229.

6. Floss, D.S.; Levy, J.G.; Levesque-Tremblay, V.; Pumplin, N.; Harrison, M.J. DELLA proteins regulate arbuscule formation in arbuscular mycorrhizal symbiosis. *Proc. Natl. Acad. Sci. U. S. A.* **2013**, *110*, E5025-E5034, doi:10.1073/pnas.1308973110.

7. Gao, L.L.; Anderson, J.P.; Klingler, J.P.; Nair, R.M.; Edwards, O.R.; Singh, K.B. Involvement of the octadecanoid pathway in bluegreen aphid resistance in *Medicago truncatula*. *Mol. Plant. Microbe Interact.* **2007**, *20*, 82-93, doi:10.1094/mpmi-20-0082.

8. Song, Y.Y.; Ye, M.; Li, C.Y.; Wang, R.L.; Wei, X.C.; Luo, S.M.; Zeng, R.S. Priming of anti-herbivore defense in tomato by arbuscular mycorrhizal fungus and involvement of the jasmonate pathway. *J. Chem. Ecol.* **2013**, *39*, 1036-1044, doi:10.1007/s10886-013-0312-1.
