# Supplementary figures and images for: Assessment of Local and Systemic Changes in Plant Gene Expression and Aphid Responses during Potato Interactions with Arbuscular Mycorrhizal Fungi and Potato Aphids
Source: Plants (Basel). 2020 Jan 9;9(1):82. doi: 10.3390/plants9010082 (PMC7020417; doi:10.3390/plants9010082)

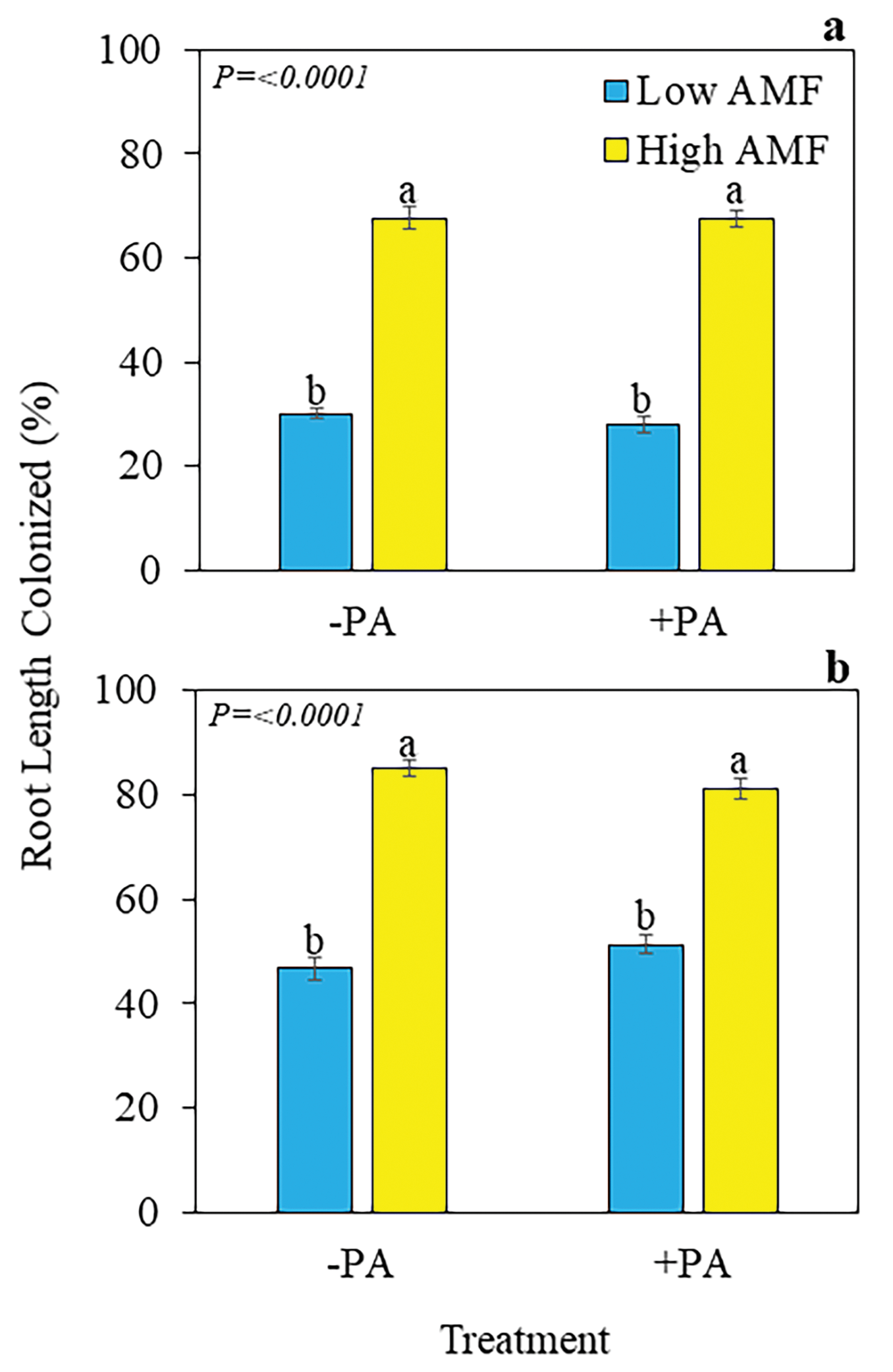

Supplement: Supplementary file 1 [file plants-09-00082-s001.zip › Revised Suppl Files/Fig. S1.tif]

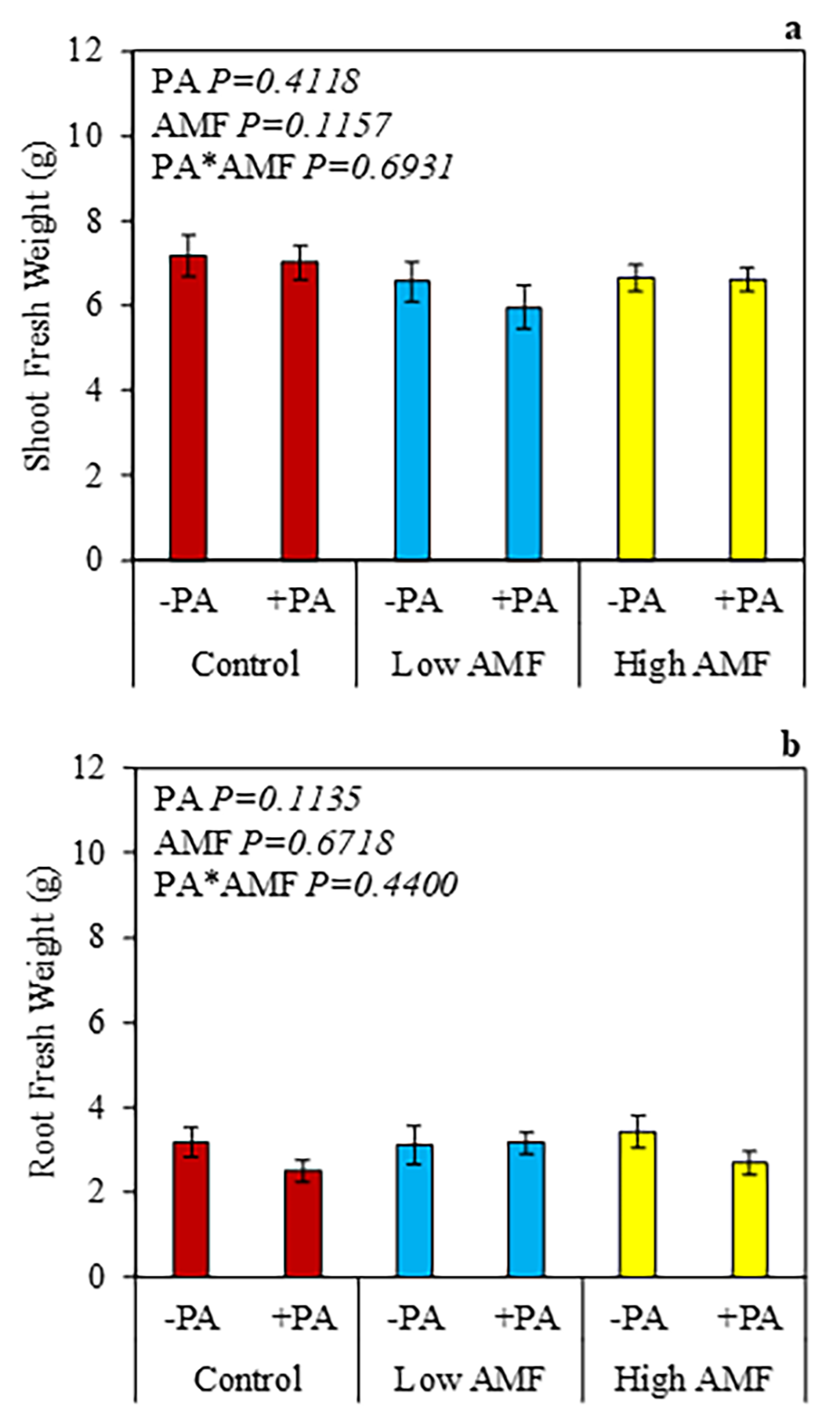

Supplement: Supplementary file 1 [file plants-09-00082-s001.zip › Revised Suppl Files/Fig. S2.tif]

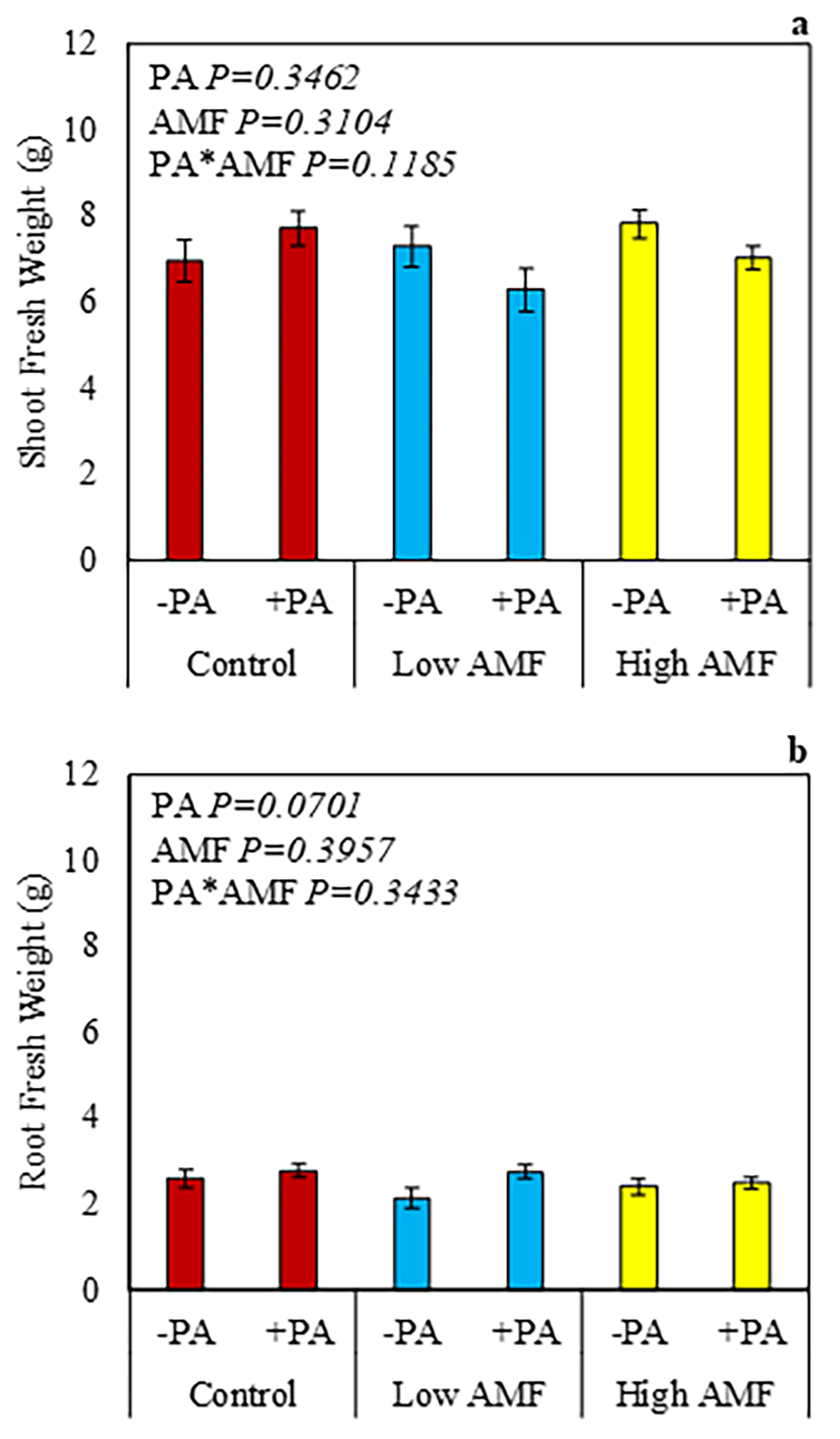

Supplement: Supplementary file 1 [file plants-09-00082-s001.zip › Revised Suppl Files/Fig. S3.tif]
